# Coupling acid catalysis and selective oxidation over MoO_3_-Fe_2_O_3_ for chemical looping oxidative dehydrogenation of propane

**DOI:** 10.1038/s41467-023-37818-w

**Published:** 2023-04-11

**Authors:** Xianhui Wang, Chunlei Pei, Zhi-Jian Zhao, Sai Chen, Xinyu Li, Jiachen Sun, Hongbo Song, Guodong Sun, Wei Wang,, Xin Chang, Xianhua Zhang, Jinlong Gong

**Affiliations:** 1 School of Chemical Engineering & Technology, Key Laboratory for Green Chemical Technology of Ministry of Education, Tianjin University, 300072 Tianjin, China; 2 Collaborative Innovation Center for Chemical Science & Engineering (Tianjin), 300072 Tianjin, China; 3 Haihe Laboratory of Sustainable Chemical Transformations, 300192 Tianjin, China; 4 Joint School of National University of Singapore and Tianjin University, International Campus of Tianjin University, 350207 Binhai New City, Fuzhou China

**Keywords:** Heterogeneous catalysis, Chemical engineering, Materials for energy and catalysis

## Abstract

Redox catalysts play a vital role in chemical looping oxidative dehydrogenation processes, which have recently been considered to be a promising prospect for propylene production. This work describes the coupling of surface acid catalysis and selective oxidation from lattice oxygen over MoO_3_-Fe_2_O_3_ redox catalysts for promoted propylene production. Atomically dispersed Mo species over γ-Fe_2_O_3_ introduce effective acid sites for the promotion of propane conversion. In addition, Mo could also regulate the lattice oxygen activity, which makes the oxygen species from the reduction of γ-Fe_2_O_3_ to Fe_3_O_4_ contribute to selectively oxidative dehydrogenation instead of over-oxidation in pristine γ-Fe_2_O_3_. The enhanced surface acidity, coupled with proper lattice oxygen activity, leads to a higher surface reaction rate and moderate oxygen diffusion rate. Consequently, this coupling strategy achieves a robust performance with 49% of propane conversion and 90% of propylene selectivity for at least 300 redox cycles and ultimately demonstrates a potential design strategy for more advanced redox catalysts.

## Abstract (web summary)

A MoO_3_-Fe_2_O_3_ redox catalyst with dispersed Mo species on γ-Fe_2_O_3_ is synthesized for the oxidative dehydrogenation of propane via chemical looping. Acid sites and lattice oxygen regulation promote the production of and selectivity for propylene.

## Introduction

Propylene is an important building block in the chemical industry^1,2^. Due to the growing gap between the demand and supply of propylene produced from steam cracking and fluidized catalytic cracking (FCC) processes, on-purpose propylene production has attracted extensive attention^3,4^. Non-oxidative propane dehydrogenation (PDH) has been implanted commercially over Pt- and Cr-based catalysts, but the propane conversion is restricted by thermodynamic equilibrium limitation^5,6^. On the contrary, oxidative dehydrogenation (ODH) with different oxidants, such as air, oxygen, N_2_O, or CO_2_, promotes propylene production. From the thermodynamic view, the oxidants consume hydrogen with a strongly exothermic reaction, which shifts the thermodynamic equilibrium toward the product of propylene^7–9^. ODH process over Cr_2_O_3_ has been extensively investigated. Ab initio multiscale kinetic study is an effective strategy to theoretically understand the reaction network and key intermediates^8,10–12^. However, the propylene selectivity should be further improved, and the participation of oxidants like O_2_ can potentially lead to explosion hazards.

Comparatively, chemical looping oxidative dehydrogenation (CL-ODH) is an effective strategy to address these challenges^13–16^. Typically, the CL-ODH process is composed of dehydrogenation and regeneration reactors. In the dehydrogenation reactor, metal oxides, defined as redox catalysts, are reduced by propane. The reduced redox catalysts are subsequently re-oxidized in the regeneration reactor under air flow. Recently, a Mo-V-O mixed oxide for CL-ODH has been reported^14^. The dual-functional Mo-V-O presented a superior propylene production rate (6.9 mol C_3_H_6_/kg-cat/h) with 89% of propylene selectivity at 500 °C using the chemical looping technology. In addition to Mo-V-O, chemical looping also boosted ethylene production with over 90% of ethylene selectivity, employing alkali metal-promoted Mn-based^15^, perovskite^13^, and NiO-based redox catalysts^16^. The activity of different oxygen species has a major influence on oxidation reactions^17,18^. Nonetheless, the volatilization and toxicity of vanadium oxide and the limited oxygen capacity of perovskite are still holdbacks.

Alternatively, economical and environmentally friendly Fe_2_O_3_ with high oxygen capacity singles out to be the desired candidate for industrial application^19–21^. Fe_2_O_3_ hitherto was widely investigated in chemical looping processes, but few of the reports illustrated the CL-ODH process over Fe_2_O_3_ due to its limited catalytic performance in alkane dehydrogenation reaction^22–24^. Moreover, Fe_2_O_3_ is inclined to over-oxidation without promoters^22,25,26^. MoO_3_ was widely studied for alkane dehydrogenation^27–32^. The introduction of MoO_3_ can be an effective strategy to graft extra active sites for propane activation. Furthermore, doping with higher valence metal cations (Mo^6+^) is an effective strategy to moderate the lattice oxygen activity^14^. Thus, Mo dopant plausibly promotes propane activation and modulates oxygen activity simultaneously. Novotný et al.^22^ prepared Fe_2_O_3_@MoO_3_ oxides for ethane CL-ODH. Surface MoO_*x*_-rich layer improved ethylene selectivity. However, ethane conversion deteriorated due to the formation of Fe_2_(MoO_4_)_3_^22^. It would be advantageous to couple the alkane activation ability from MoO_3_ and the oxidation capacity from Fe_2_O_3_ for chemical looping oxidative dehydrogenation of propane.

Herein, we demonstrate the promotion of Mo dopant in Fe_2_O_3_-based redox catalysts for propane conversion via CL-ODH. Coupling the acid-catalytic ability from Mo dopant and mild lattice oxygen, MoO_3_-Fe_2_O_3_ presented a higher surface exchange coefficient (1.6 × 10^−4^ cm/s) and moderate oxygen diffusion coefficient (6.7 × 10^−9^ cm^2^/s). Consequently, propylene productivity (9.6 mol C_3_H_6_/kg-cat/h) was greatly improved. As confirmed by aberration-corrected high-angle annular dark-field scanning transmission electron microscope (AC-HAADF-STEM), extended X-ray absorption fine structure (EXAFS), Raman, X-ray diffraction (XRD), and X-ray photoelectron spectroscopy (XPS), Mo atoms were atomically dispersed and enriched on the surface of as-synthesized redox catalysts. Subsequently, surface-enriched Mo cations induced great modification in acid properties, evidenced by NH_3_ temperature-programmed desorption (NH_3_-TPD) and diffused reflectance infrared Fourier transform spectroscopy (NH_3_-DRIFTS). C_3_H_8_-D_2_ temperature-programmed surface reaction (C_3_H_8_-D_2_-TPSR) indicated that extra acid sites (Mo-OH) derived from isolated Mo atoms drastically promoted propane activation. Meanwhile, the Mo dopant effectively modified the lattice oxygen activity of the γ-Fe_2_O_3_ matrix. The dynamic change regarding the inhibited lattice oxygen activity and alleviated oxygen out-diffusion was systematically studied by H_2_ and C_3_H_8_ temperature-programmed reduction (H_2_-TPR and C_3_H_8_-TPR), in situ DRIFTS, in situ Raman, and in situ XRD. Combining the kinetics experiments, thermal gravity (TG), C_3_H_8_ isothermal pulse and products distribution with time on stream, the coupling between acid catalysis, and selective oxidation was further confirmed.

## Results

### Dehydrogenation performance and redox stability test

In this work, a series of Al_2_O_3_-supported MoO_3_-Fe_2_O_3_ with different molar ratios of Fe to Mo (1MoxFeAl, x = 15, 12, 9, 6) were synthesized via the conventional impregnation method. FeAl and MoAl were also prepared as references. See the “Methods” section for details.

**Fig. 1:**
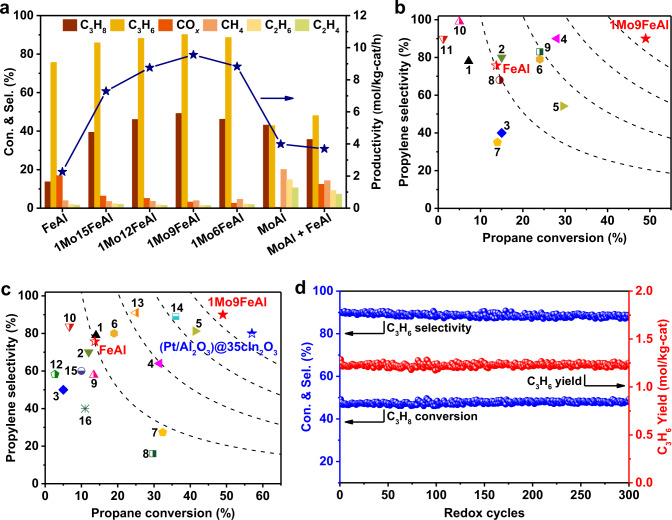
Catalytic performance and stability test. (**a**) Products distribution over different redox catalysts at the fourth minute, FeAl + MoAl represents the physical mixture of MoAl and FeAl. Comparison of 1Mo9FeAl with the catalytic performance of reported (**b**) Fe-based catalysts and (**c**) ODH catalysts. All referenced catalysts and citations are listed in Supplementary Tables 1 and 2. (**d**) Redox stability of 1Mo9FeAl in chemical looping oxidative dehydrogenation of propane. Reaction conditions: 570 °C, 0.14 MPa, GHSV = 3000 h^−1^, 0.5 g sample, volumetric C_3_H_8_/N_2_ ratio = 4:17. The identification of optimal reaction conditions is shown in Supplementary Fig. 1.

As shown in Fig. 1a, Mo had a prominent influence on the performance of Fe_2_O_3_-based redox catalysts. At the oxidative dehydrogenation stage with minor CO_*x*_ (Supplementary Fig. 2)^14^, 1Mo9FeAl can reach up to 49% of propane conversion, 90% of propylene selectivity, and 98% of carbon balance or 9.6 mol C_3_H_6_/kg-cat/h for transient production rate at the fourth minute. The integral conversion and selectivity were determined to be 49% and 89% within 10 minutes (Supplementary Figs. 3 and 4). As summarized in Fig. 1b, c, the optimal 1Mo9FeAl presented comparable performance with that of (Pt/Al_2_O_3_)@35cIn_2_O_3_^7^. It also outperformed state-of-the-art Fe-based and other reported ODH catalysts. Comparatively, FeAl showed inferior performance in both propane conversion (14%) and propylene selectivity (76%), while the MoAl catalyst tended to cause cracking and carbon deposition. Therefore, more coke was burnt for the spent MoAl catalyst in comparison to 1Mo9FeAl in O_2_ temperature-programmed oxidation (O_2_-TPO) results (Supplementary Fig. 5). The physical mixture of MoAl and FeAl also presented inferior propylene selectivity, highlighting the formation of mixed metal oxides (MoO_3_-Fe_2_O_3_). Meanwhile, the pre-reduced 1Mo9FeAl redox catalyst under H_2_ flow (1Mo9FeAl-red) presented lower activity and propylene selectivity. The inferior performance emphasized the promotional effects of lattice oxygen for propylene production (Supplementary Fig. 6).

Cyclic stability is a significant evaluation criterion in the CL-ODH process. Redox experiments were carried out over 1Mo9FeAl (Fig. 1d). Propane conversion, propylene selectivity, and integral propylene yield kept stable for 300 redox cycles for 1Mo9FeAl, when the optimal regeneration time was 10 minutes (Supplementary Figs. 7 and 8)^33–37^. In accordance with the robust redox stability, the XRD patterns and crystal sizes were consistent for fresh and regenerated redox catalysts (Supplementary Fig. 9 and Supplementary Table 3). Meanwhile, the molar ratio of Fe to Mo (Supplementary Fig. 10 and Supplementary Table 4) was constant for regenerated 1Mo9FeAl. The stable structure after multiple dehydrogenation-regeneration cycles led to stable performance. In summary, Mo dopant promoted both propane conversion and propylene selectivity in comparison to that of pristine FeAl. The improvement in propane conversion and propylene selectivity should be ascribed to the promoted propane activation and modification in lattice oxygen activity, respectively, and it will be discussed later. Meanwhile, the optimal 1Mo9FeAl redox catalyst presented a robust performance in the stability test, and the stable structure of the redox catalyst was favorable for its further application.

### Textural properties

The textural properties of the redox catalysts were primarily characterized to illustrate the fine structure of 1MoxFeAl. The redox catalysts experienced one dehydrogenation-regeneration cycle and demonstrated a phase transformation from α-Fe_2_O_3_ (JCPDS 33-0664, hematite) to γ-Fe_2_O_3_ (JCPDS 39-1346, maghemite) according to XRD measurement^36,37^, which were adopted for in-depth study (Supplementary Fig. 11). See the Supplementary Information for details. Basically, the Mo dopant had a minor effect on the surface area, pore volume, pore size distributions, and morphology because of the high loading of γ-Fe_2_O_3_ (Supplementary Figs. 12 and 13, Supplementary Table 5).

**Fig. 2:**
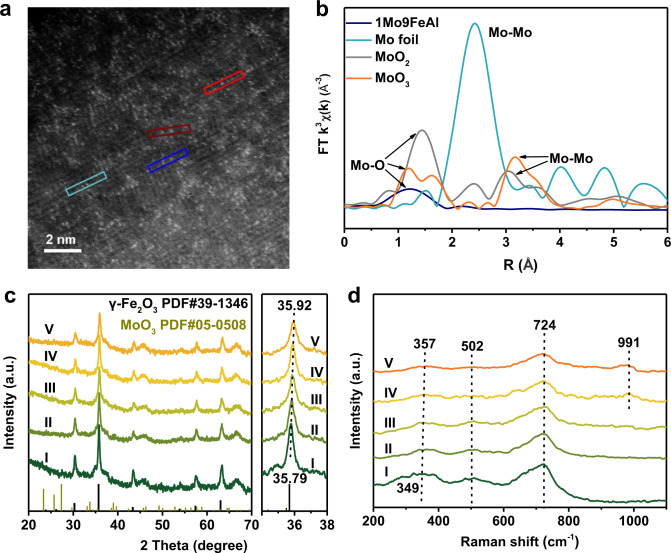
Morphology and textural properties of MoO_3_-Fe_2_O_3_ redox catalysts. (**a**) AC-HAADF-STEM image of 1Mo9FeAl. (**b**) EXAFS spectra at Mo K-edge for 1Mo9FeAl, Mo foil, MoO_2_, and MoO_3_. XRD patterns (**c**) and Raman spectra (**d**) of as-prepared redox catalysts. I, II, III, IV, and V are for FeAl, 1Mo15FeAl, 1Mo12FeAl, 1Mo9FeAl and 1Mo6FeAl, respectively. The a.u. stands for arbitrary units.

AC-HAADF-STEM was employed to directly distinguish the location of Mo cations (Fig. 2a, Supplementary Fig. 14). Bright spots corresponding to isolated Mo atoms were detected in the Fe_2_O_3_ crystallites, despite the high density of Mo atoms. The isolation of Mo atoms was beneficial for propylene production since side reactions, such as cracking, were structure sensitive. EXAFS was further performed to confirm the isolation of Mo atoms. As shown in Fig. 2b and Supplementary Fig. 15, 1Mo9FeAl presented Mo-O coordination at around 1.80 Å without the appearance of Mo-Mo scatting paths, and the coordination number (CN) for Mo-O was determined to be about 4 (Supplementary Table 6)^38,39^. The insignificant contribution of Mo-Mo scattering paths to the fitting data of 1Mo9FeAl from EXAFS results suggested the high dispersion of Mo atoms. Energy dispersive spectrometry (EDS) line scan in Supplementary Fig. 16 demonstrated that Mo atoms were in good coordination with the distribution of Fe element, indicating that Mo cations were primarily dispersed on Fe_2_O_3_ instead of Al_2_O_3_. Further increasing Mo content, Mo ensembles inevitably appeared in 1Mo6FeAl (Supplementary Fig. 17), which would catalyze the structure-sensitive side reactions and cause inferior propylene selectivity.

Due to the atomic dispersion of Mo atoms, FeAl and 1MoxFeAl presented typical characteristic peaks of γ-Fe_2_O_3_ without the appearance of MoO_3_ (Fig. 2c)^36,37^. The characteristic peaks were broader and weaker with increasing Mo content, implying the high dispersion of Mo cations. Furthermore, the characteristic peaks slightly shifted to a higher diffraction angle because of the smaller ionic radius of Mo^6+^ (0.59 Å) compared with that of Fe^3+^ (0.65 Å) (Supplementary Table 7)^40^. The linear relationship between the peak shift and Mo contents indicated that Mo atoms were inserted into the γ-Fe_2_O_3_ matrix (Supplementary Fig. 18a). By contrast, the physical mixture of FeAl and MoAl exhibited identical characteristic peaks with that of FeAl (Supplementary Fig. 18b). Furthering increasing Mo content, Fe_2_(MoO_4_)_3_ inevitably appeared for 1Mo3FeAl redox catalyst in the XRD pattern (Supplementary Fig. 19). As to MoAl catalyst (Supplementary Fig. 20), diffraction peaks ascribed to MoO_3_ were not detected^41^.

Raman spectroscopy was also conducted to demonstrate the change in textual properties of 1MoxFeAl with Mo addition. FeAl and 1MoxFeAl exhibited exclusive resonant Raman peaks at 349–357, 502, 724 cm^−1^, corresponding to *T*_2_, *E*, *A*_1_ vibrational modes of γ-Fe_2_O_3_ (Fig. 2d)^42–44^. Identical to the XRD patterns, these peaks became weaker and broader, and the peak at 349 cm^−1^ shifted to a higher Raman shift with enhanced Mo content. Meanwhile, Raman spectra of 1Mo6FeAl and 1Mo9FeAl presented a specific vibrational peak at 992 cm^−1^ for Mo=O because of the distortion of [MoO_6_] units and the intensity increased with Mo concentration^25,45^. For 1Mo3FeAl, it exhibited peaks assigned to Fe_2_(MoO_4_)_3_ at 965 and 780 cm^−1^ (Supplementary Fig. 21), which was in accordance with the XRD patterns. Moreover, by comparison, Raman peaks at 815 and 875 cm^−1^ corresponding to Al_2_(MoO_4_)_3_ and peak at 965 cm^−1^ ascribed to polymeric MoO_*x*_ were presented on MoAl and the physical mixture of MoAl and FeAl, but absent for 1Mo9FeAl in Supplementary Fig. 22^31,32,46^. The difference in Raman spectra between FeAl, MoAl, and 1Mo9FeAl redox catalysts further demonstrated that Mo cations were located on γ-Fe_2_O_3_ instead of Al_2_O_3_.

Based on the above mentioned AC-HAADF-STEM, EXAFS, XRD, and Raman results, atomically dispersed Mo cations should be incorporated into the lattice of γ-Fe_2_O_3_ rather than dispersed on Al_2_O_3_ support. The isolation of Mo cations by surrounding γ-Fe_2_O_3_ matrix inhibited the appearance of Mo ensembles, which alleviated the structure-sensitive side reactions. However, further increasing Mo content, Mo ensembles and Fe_2_(MoO_4_)_3_ unavoidably formed in 1Mo6FeAl and 1Mo3FeAl redox catalyst, respectively. Generally, γ-Fe_2_O_3_ acted well as a host for the atomic dispersion of Mo atoms.

### Mo accelerated surface reaction

The surface composition and chemical state of the redox catalysts were directly related to the improved performance for 1MoxFeAl. XPS experiments were performed accordingly.

**Fig. 3:**
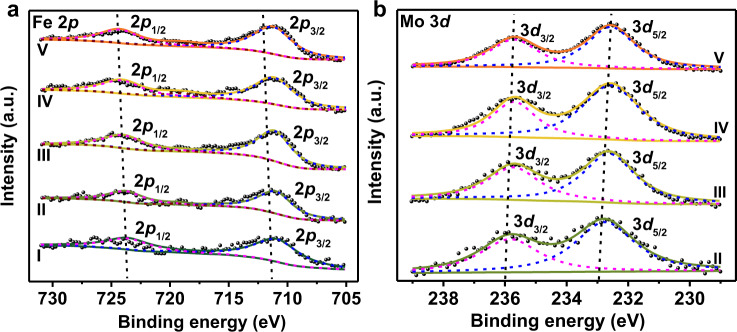
XPS measurements of MoO_3_-Fe_2_O_3_ redox catalysts. XPS spectra for (**a**) Fe 2*p*, (**b**) Mo 3*d* of as-prepared redox catalysts. I, II, III, IV, and V are for FeAl, 1Mo15FeAl, 1Mo12FeAl, 1Mo9FeAl, and 1Mo6FeAl, respectively. The a.u. stands for arbitrary units.

Figure 3a and Supplementary Fig. 23 presented the XPS spectra of Fe 2*p*, and the peaks were centered at 710.9 and 724.3 eV, suggesting the emergence of separate Fe^3+^^47,48^. Trivalent Fe cation was further verified by Fe K-edge X-ray absorption near-edge structure (XANES) spectra. FeAl and 1Mo9FeAl, with reference to γ-Fe_2_O_3_, presented similar curves in Fe K-edge XANES spectra, indicating the exclusive existence of Fe^3+^ in FeAl and 1Mo9FeAl redox catalyst (Supplementary Fig. 24). XPS spectra of Mo 3*d* shown in Fig. 3b demonstrated the exclusive existence of Mo^6+^ species^27,29^. In accordance with previous literature^22^, Fe^3+^ shifted to higher binding energy, while Mo^6+^ shifted toward a lower value. However, Mo 3*d* spectra of MoAl shifted to a much higher binding energy. The back shift in binding energy for MoAl suggested the intimate interaction between Mo and γ-Fe_2_O_3_ in 1MoxFeAl redox catalysts, supporting the peak shift in the XRD patterns and Raman spectra (Supplementary Fig. 25). Moreover, the derived surface composition shown in Supplementary Table 8 demonstrated that Mo cations took up a higher portion over the surface of 1MoxFeAl. The enrichment of Mo was beneficial for propane activation.

**Fig. 4:**
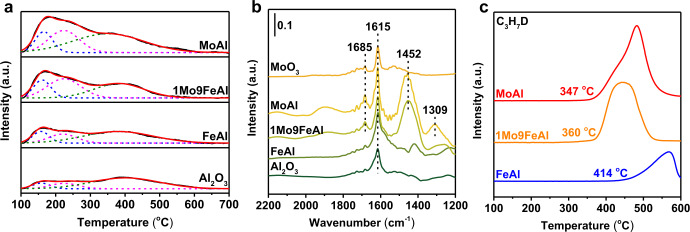
Modification on surface acid properties and its sequent promotion on propane activation. (**a**) NH_3_-TPD profiles of Al_2_O_3_, FeAl, 1Mo9FeAl, and MoAl. NH_3_-TPD was performed from 100 to 700 °C with a rate of 10 °C/min after being pretreated in a flow of 5 vol% NH_3_ in He (20 mL/min) for 1 h at 100 °C. (**b**) NH_3_-DRIFTS spectra of Al_2_O_3_, FeAl, 1Mo9FeAl, MoAl, and MoO_3_. (**c**) Signal of C_3_H_7_D during C_3_H_8_-D_2_-TPSR over FeAl, 1Mo9FeAl, and MoAl. The C_3_H_8_-D_2_-TPSR experiments were conducted from 100 to 600 °C with a rate of 10 °C/min in a mixture of 5 vol% C_3_H_8_ and 5 vol% D_2_ in He (10 mL/min). The a.u. stands for arbitrary units.

Obviously, Mo addition had a pronounced effect on the surface properties of redox catalysts. According to previous report^28^, the surface acid properties directly affected the dehydrogenation reaction over Mo-based catalysts. NH_3_-TPD was preliminary performed to detect the surface acid concentration. γ-Fe_2_O_3_ induced great modification in weak and medium acid sites in comparison to that of Al_2_O_3_. Twice the amount of weak and medium acid sites appeared for FeAl, while strong acid sites remained constant (Fig. 4a, Supplementary Table 9)^49,50^. The introduction of Mo further increased acid site density. Especially, 1Mo9FeAl presented four times the amount of medium acid sites. However, MoAl presented twice the amount of strong acid sites, leading to severe side reactions and coke formation.

In addition to acid density, the introduction of Mo atoms also induced a significant modification on surface Lewis/Brønsted acid properties. As shown in Fig. 4b, Al_2_O_3_ and FeAl exhibited a single NH_3_-adsorption peak at 1615 cm^−1^, which was ascribed to Lewis acid sites, while new peaks at 1685 and 1452 cm^−1^ ascribed to Brønsted acid emerged in 1Mo9FeAl and MoAl^51–53^. Moreover, a specific peak at 1309 cm^−1^ assigned to Lewis acid sites from the interaction of MoO species and Al_2_O_3_ support appeared for MoAl, which, however, was absent for 1Mo9FeAl^54,55^. NH_3_-DRIFTS for the physical mixture of MoO_3_ and γ-Fe_2_O_3_ was also tested for comparison. The physical mixture presented NH_3_-adsorption peaks at 1450 and 1683 cm^−1^ for Brønsted acid despite that bulk MoO_3_ and γ-Fe_2_O_3_ showed a separate Lewis acid at 1615 cm^−1^ (Supplementary Fig. 26). Considering the XPS results that Fe and Mo cations were exclusively trivalent and hexavalent, -OH with negative electrons should be introduced on the charge-balancing sites created by the atomically Mo doping in Fe_2_O_3_ lattice to form Mo-OH. Mo-OH was then titrated as Brønsted acid sites in NH_3_-DRIFTS experiments^56,57^. The appearance of Brønsted acid for the physical mixture of MoO_3_ and γ-Fe_2_O_3_ at 1450 cm^−1^ and the absence of Lewis acid site at 1309 cm^−1^ for 1Mo9FeAl further demonstrated that Mo atoms should be located over Fe_2_O_3_ for 1Mo9FeAl. Combining the above NH_3_-DRIFTS results and the inferior propylene selectivity over the physical mixture of MoAl and FeAl, Mo-OH derived from Mo atoms isolated by γ-Fe_2_O_3_ should be responsible for the selective propylene production in 1Mo9FeAl.

The enrichment of Mo on the surface of 1MoxFeAl redox catalysts and its modulation of acid properties significantly promoted propane activation. As shown in the C_3_H_8_-D_2_-TPSR results (Fig. 4c), propane was activated at 414 °C over FeAl redox catalyst, while the activation temperature was decreased to 360 °C under the promotion of Mo dopant. MoAl exhibited the best activation ability at 347 °C. The promoted propane activation conformed to the difference in catalytic performance that 1Mo9FeAl and MoAl presented a superior performance in propane conversion. In the dehydrogenation process, the stable acid properties (Supplementary Fig. 27) and Mo^4+^/Mo^6+^ ratio (11.7%, Supplementary Fig. 28a and Supplementary Table 10) provided constant catalytic sites for propane conversion. The consecutive out-diffusion of lattice oxygen from the reduction of Fe^3+^ to Fe^2+^ was the primary cause for the stabilization of Mo valence states (Supplementary Fig. 29). By contrast, much more Mo^6+^ was reduced to Mo^4+^ for the MoAl catalyst (23.2%) without consecutively out-diffused lattice oxygen (Supplementary Fig. 28b and Supplementary Table 11).

In summary, the introduction of the Mo atom had a prominent effect on the surface properties of the redox catalyst. Based on XPS spectra, Mo atoms were enriched on the surface of 1MoxFeAl redox catalysts. Meanwhile, surface-enriched Mo atoms significantly modified the acid properties of the redox catalysts. The Mo-OH derived from the Mo single atoms segregated by the γ-Fe_2_O_3_ matrix was the active site for propane activation. Because of the surface-enriched Mo cations and its modulation on acid properties, 1Mo9FeAl redox catalyst exhibited much better capacity in propane activation, as shown in the C_3_H_8_-D_2_-TPSR results. In the dehydrogenation process, γ-Fe_2_O_3_ was gradually reduced and provided consecutively out-diffused lattice oxygen to stabilize the Mo valence state and consume the abstracted H species, promoting propylene production.

### Mo modulated oxygen evolution

In addition to the accelerated surface reaction, the improved propylene selectivity and deteriorated CO_*x*_ content were directly related to the moderated lattice oxygen activity.

**Fig. 5:**
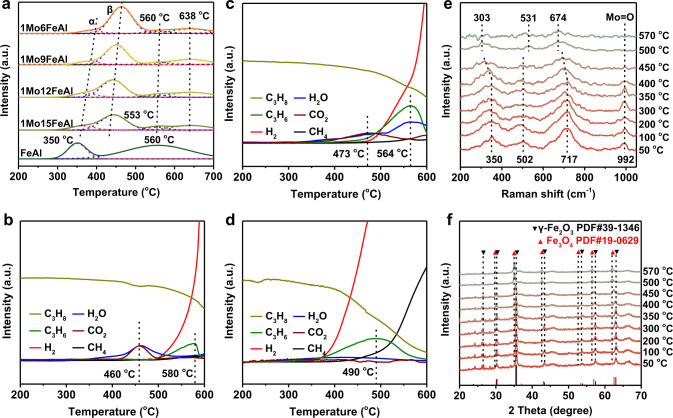
Modulation of overactive lattice oxygen species for MoO_3_-Fe_2_O_3_ redox catalysts. (**a**) H_2_-TPR profiles of different redox catalysts ramping from 200 to 700 °C with a rate of 10 °C/min in a mixture of 10 vol% H_2_ in Ar (30 mL/min). C_3_H_8_-TPR mass spectra of (**b**) FeAl, (**c**) 1Mo9FeAl, (**d**) MoAl. The C_3_H_8_-TPR experiments were conducted from 200 to 600 °C with a rate of 10 °C/min in a mixture of 15 vol% C_3_H_8_ in He (10 mL/min). (**e**) In situ Raman spectra and (**f**) in situ XRD patterns of 1Mo9FeAl under the flow of 15 vol% C_3_H_8_ in He (20 mL/min) at different temperatures. The a.u. stands for arbitrary units.

H_2_-TPR is a routine technique for evaluating the activity of different oxygen species. As shown in Fig. 5a, FeAl sample presented two typical reduction peaks as pure γ-Fe_2_O_3_ at 350 °C and 560 °C respectively. The reduction peak at 350 °C was corresponding to the reduction of γ-Fe_2_O_3_ to Fe_3_O_4_, while the peak at 560 °C was the integrated results for the reduction of Fe_3_O_4_ to FeO and FeO to Fe^58–61^. For 1MoxFeAl redox catalysts, the temperature for the first reduction peak was gradually increased with Mo content, and the asymmetric peak could be resolved into pairs (O_α_ and O_β_). Considering the surface enrichment of Mo atoms and the low reduction temperature for MoAl (Supplementary Fig. 30), O_α_ was ascribed to the reduction of surface-enriched MoO_*x*_, while O_β_ was corresponding to the reduction of γ-Fe_2_O_3_ to Fe_3_O_4_. Based on the quantitative reduction temperature from H_2_-TPR (Supplementary Table 12), the reduction temperature for γ-Fe_2_O_3_ to Fe_3_O_4_ in 1Mo9FeAl was increased by 101 °C compared with that of FeAl.

The distinction of different oxygen species (O_α_ and O_β_) was directly related to the products distribution in C_3_H_8_-TPR results. For FeAl, oxygen species from the reduction of γ-Fe_2_O_3_ to Fe_3_O_4_ over-oxidized propylene to CO_2_ and H_2_O at 460 °C (Fig. 5b). Further increasing reduction temperature, propylene intensity reached a maximum value at 580 °C without CO_2_ and H_2_O, indicating the occurrence of non-oxidative dehydrogenation. However, the oxidative dehydrogenation stage vanished for FeAl. Comparatively, 1Mo9FeAl presented a different C_3_H_8_-TPR result (Fig. 5c). O_α_ devoted to over-oxidation with single pairs of CO_2_ and H_2_O at 473 °C, while O_β_ contributed to oxidative dehydrogenation with the production of C_3_H_6_ and H_2_O at 564 °C. The increase in reduction temperature from γ-Fe_2_O_3_ to Fe_3_O_4_ between FeAl and 1MoxFeAl in H_2_-TPR results indicated the modification in lattice oxygen activity. Consequently, the original overactive oxygen species (γ-Fe_2_O_3_ to Fe_3_O_4_) in FeAl turned to be the moderate lattice oxygen (O_β_) in 1Mo9FeAl for selectively oxidative dehydrogenation reaction. As to MoAl, propylene and H_2_ appeared simultaneously in Fig. 5d with much lower CO_2_ and H_2_O signal intensity, indicating that the MoAl catalyst was primarily devoted to non-oxidative dehydrogenation.

To further examine the evolution of different oxygen species under reaction conditions, in situ Raman measurements were operated (Fig. 5e). At 50 °C, 1Mo9FeAl presented typical Raman peaks as γ-Fe_2_O_3_^42–44^. Peaks at 350 and 717 cm^−1^ shifted to the lower Raman shift with the consumption of lattice oxygen at dehydrogenation temperatures above 400 °C. Fe_3_O_4_ was ultimately formed at 500 °C with the appearance of new peaks at 303 and 674 cm^−1^, indicating the controllable release of lattice oxygen^62–65^. Meanwhile, the complete disappearance of the peak located at 1409 cm^−1^ at 500 °C was also strong evidence for the phase transformation from γ-Fe_2_O_3_ to Fe_3_O_4_ (Supplementary Fig. 31). Besides the consumption of lattice oxygen from the reduction of γ-Fe_2_O_3_ to Fe_3_O_4_, surface Mo=O was also depleted at 400 °C. The consumption of Mo=O in Raman spectra was identical to the in situ DRIFTS results that Mo=O presented a negative peak at 400 °C (Supplementary Fig. 32)^66,67^. However, the consumed Mo=O can be replenished by the consecutively out-diffused lattice oxygen after regenerated at 570 °C under N_2_ flow (Supplementary Fig. 33), which was in accordance with the stable Mo valence states in XPS results for spent 1Mo9FeAl. Comparatively, phase transformation took place at 400 °C for FeAl, and the consumed lattice oxygen was supposed to deeply crack the produced propylene (Supplementary Fig. 31).

In situ XRD experiments presented comparable results to in situ Raman. 1Mo9FeAl and FeAl exhibited typical diffraction peaks corresponding to γ-Fe_2_O_3_ at dehydrogenation temperatures lower than 350 °C (Fig. 5f and Supplementary Fig. 34)^36,37^. With the depletion of lattice oxygen, a phase transformation from γ-Fe_2_O_3_ to Fe_3_O_4_ suddenly occurred at 400 °C for FeAl^33–35^. However, due to the moderate oxygen activity, the phase transformation terminated at 500 °C for 1Mo9FeAl, which was in correspondence with that in situ Raman spectra. Moreover, 1Mo9FeAl exhibited a superior ability for controllable oxygen release and prolonged oxidative dehydrogenation stage from the isothermal XRD patterns (Supplementary Fig. 35).

In summary, the Mo dopant effectively regulated the lattice oxygen activity in γ-Fe_2_O_3_. For FeAl, γ-Fe_2_O_3_ was reduced to Fe_3_O_4_ at 350 °C, and this specific oxygen species devoted to the over-oxidation of propylene. Oxygen species ascribed to the reduction of Fe_3_O_4_ was inert in oxidative dehydrogenation because of the high reduction temperature. With the addition of Mo atoms, MoO_*x*_ enriched on the surface was reduced at 400 °C (O_α_) and contributed to over-oxidation, while the temperature for the reduction of γ-Fe_2_O_3_ to Fe_3_O_4_ for 1Mo9FeAl (O_β_) was increased by 101 °C, which was then devoted to oxidative dehydrogenation. The increase in reduction temperature between FeAl and 1MoxFeAl in H_2_-TPR results induced a great modification in lattice oxygen activity. Consequently, the original overactive oxygen species (γ-Fe_2_O_3_ to Fe_3_O_4_) in FeAl devoted to selectively oxidative dehydrogenation reaction over 1Mo9FeAl.

### Coupling effects of surface acid catalysis and selective oxidation

Based on the above catalytic performance and characterization results, isolated Mo atoms had a prominent influence on both the surface catalytic process and lattice oxygen activity for oxidative dehydrogenation. Atomically dispersed Mo atoms introduced abundant acid sites (Mo-OH) for propane conversion and passivated the lattice oxygen activity, matching the surface reaction and bulk oxygen diffusion to improve propylene selectivity. The coupling between surface acid catalysis and selective oxidation was further studied by kinetics experiments^68,69^. The detailed calculation for the removal of external and internal mass transfer limitations can be found in the “Methods” section and Supplementary Table 13. The oxygen surface exchange coefficient, *k*, and bulk diffusion coefficient, *D*, shown in Supplementary Table 14, were calculated to quantify the surface reaction rate and lattice oxygen diffusion rate. Due to the promotion of surface acid sites, the surface reaction rate coefficient for 1Mo9FeAl (1.6 × 10^−4^ cm/s) was significantly accelerated, about an order of magnitude higher than that of FeAl (1.2 × 10^−5^ cm/s). A high surface reaction rate coefficient resulted in much higher propane conversion, as shown in Fig. 1. Meanwhile, Mo dopant moderated lattice oxygen diffusion, leading to lower *D* value for 1Mo9FeAl (6.7 × 10^−9^ cm^2^/s) in comparison to that of FeAl (2.5 × 10^−8^ cm^2^/s), which significantly inhibited the over-oxidation and extended the oxidative dehydrogenation. Thermal gravity results in Supplementary Fig. 36 demonstrated comparable results. 1Mo9FeAl presented a slower oxygen release rate, which was in correspondence with the decreased *D* value. Furthermore, oxygen consumption was negligible for 1Mo9FeAl under C_3_H_6_ flow, while the oxygen consumption rate was twice for FeAl in C_3_H_6_ flow than in C_3_H_8_ flow. The contrast in lattice oxygen consumption rate under C_3_H_6_ flow between FeAl and 1Mo9FeAl further confirmed the superior propylene selectivity over 1Mo9FeAl redox catalyst.

Kinetics results were proved by the instantaneous composition in the outlet gas at different reaction times. Based on the CO_*x*_ concentration, the dehydrogenation progress was divided into three characteristic stages, over-oxidation, oxidative dehydrogenation, and non-oxidative dehydrogenation (Supplementary Fig. 37)^14^. The partition of the three distinctive regions was further proved by the propane isothermal reaction (Supplementary Fig. 38). Apparently, much more CO_*x*_ appeared in the exhausted gas of FeAl, and the over-oxidation stage was sustained for about 5 minutes. Furthermore, due to the high oxygen diffusion rate coefficient, or greater *D* value, the oxidative dehydrogenation region lasted for only 2 minutes. By contrast, 1Mo9FeAl presented satisfied CO_*x*_ selectivity at the second minute, and the oxidative dehydrogenation region lasted for at least 7 minutes, indicating moderate oxygen release or lower *D* value. Besides the prolonged oxidative dehydrogenation stage, the drastically improved propane conversion should be solid evidence for the increase in the *k* value. In summary, coupling the improved *k*, derived from enriched surface acid sites (Mo-OH) and decreased *D*, ascribed to the passivation in the out-diffusion of lattice oxygen, 1Mo9FeAl demonstrated good performance in chemical looping oxidative dehydrogenation of propane.

**Fig. 6:**
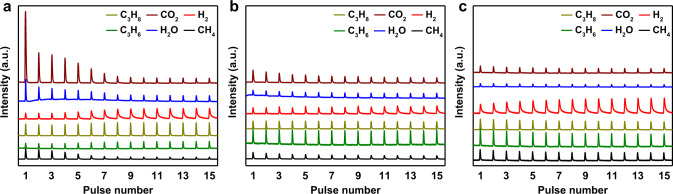
C_3_H_8_ pulses over MoO_3_-Fe_2_O_3_ redox catalysts. Mass spectra during C_3_H_8_ pulses over **a** FeAl, **b** 1Mo9FeAl, and **c** MoAl; 0.5 mL of C_3_H_8_ was injected into the reactor for each pulse at 570 °C. The a.u. stands for arbitrary units.

C_3_H_8_ isothermal pulse experiments were further performed to confirm the instantaneous composition of different products. As shown in Fig. 6a, FeAl was inclined to fully oxidize propylene in first several pulses, and the signal for H_2_O and CO_2_ was much higher than that of 1Mo9FeAl and MoAl. Consequently, it demonstrated 31% of CO_*x*_ selectivity in the first minute. In comparison to FeAl, 1Mo9FeAl could efficiently catalyze oxidative dehydrogenation (Fig. 6b), coupling surface acid catalysis and selective oxidation. 1Mo9FeAl presented higher propylene signal intensity, while the signal intensity for propane and CO_2_ was much lower in the first few C_3_H_8_ pulses. Meanwhile, the signal for H_2_, H_2_O, and C_3_H_6_ was rather stable with time on the stream, indicating the preservation of the oxidative dehydrogenation stage. For the MoAl sample (Fig. 6c), the H_2_ signal from non-oxidative dehydrogenation contributed dominantly to the exhausted gas. In accordance with the pulse experiments, H_2_O/H_2_ ratios over FeAl and 1Mo9FeAl also demonstrated the promotion of surface acid catalysis and oxidative dehydrogenation. As shown in Supplementary Fig. 39, FeAl presented a much higher H_2_O/H_2_ ratios from over-oxidation at the first few minutes. However, the H_2_O/H_2_ ratios were deteriorated in comparison to 1Mo9FeAl with the rapid depletion of lattice oxygen. For 1Mo9FeAl, O_β_ dominantly contributed to the selective oxidation, resulting in a stable H_2_O/H_2_ ratio and prolonged oxidative dehydrogenation period. Consequently, 46% of propylene was produced from the selective oxidative reaction in the CL-ODH process for 1Mo9FeAl at the fourth minute.

## Discussion

An efficient MoO_3_-Fe_2_O_3_ redox catalyst was designed for chemical looping oxidative dehydrogenation of propane. 1Mo9FeAl exhibited a superior performance with 49% of propane conversion and 90% of propylene selectivity for at least 300 cycles. Basically, atomically dispersed Mo cations enriched on the surface of 1Mo9FeAl redox catalyst introduced extra acid sites (Mo-OH), promoting propane conversion. Meanwhile, Mo cations effectively modulated lattice oxygen activity and out-diffusion rate, making the pristine over-reactive oxygen species available for selectively oxidative dehydrogenation. Due to the enhancement in surface acid sites and lattice oxygen modification upon isolated Mo doping, propylene production was significantly improved, coupling surface acid catalysis and selective oxidation. This study demonstrates the effects of surface acidity and lattice oxygen on selective dehydrogenation in chemical looping processes.

## Methods

### Catalysts preparation

#### Materials

Fe(NO_3_)_3_·9H_2_O was purchased from Aladdin biological technology Co., Ltd. (Shanghai, China). (NH_4_)_6_Mo_7_O_24_·4H_2_O was purchased from Tianjin yuanli chemical reagent Co., Ltd. (Tianjin, China). γ-Al_2_O_3_ was purchased from Adamas-beta reagent Co., Ltd. (Shanghai, China)

#### Synthesis of **FeAl**

Fe_2_O_3_/Al_2_O_3_ redox catalyst was defined as FeAl. FeAl was synthesized by a facile impregnation method, where γ-Al_2_O_3_ was employed as support, and Fe_2_O_3_ loading was fixed at 40 wt%. Typically, 3.367 g of Fe(NO_3_)_3_·9H_2_O and 1 mL of deionized water were loaded in a 10-mL disposable centrifuge tube. Fe(NO_3_)_3_·9H_2_O was dissolved in deionized water under ultrasound treatment for 1 h, forming a brown solution. The solution was then added to 1 g of γ-Al_2_O_3_ powder. The mixture was shaken on an oscillator and treated under ultrasound treatment for another 1 h. After impregnation, the brown slurry was dried at 100 °C for 12 h and then calcinated at 600 °C in a muffle furnace for 6 h.

#### Synthesis of **1MoxFeAl**

Mo-doped Fe_2_O_3_/Al_2_O_3_ redox catalysts were defined as 1MoxFeAl, where x was the molar ratio of Fe to Mo (Fe/Mo, x = 6, 9, 12, 15). 1MoxFeAl was prepared by a simple co-impregnation process, where Fe_2_O_3_ loading was fixed at 40 wt%, while the amount of Mo was dependent on x value. Consequently, 0.2452, 0.1635, 0.1226, and 0.0981 g of (NH_4_)_6_Mo_7_O_24_·4H_2_O (precursor of MoO_3_) was added for the preparation of 1Mo6FeAl, 1Mo9FeAl, 1Mo12FeAl, and 1Mo15FeAl respectively. Typically, 3.367 g of Fe(NO_3_)_3_·9H_2_O and the required amount of (NH_4_)_6_Mo_7_O_24_·4H_2_O based on x value were loaded in a 10-mL disposable centrifuge tube in sequence. The mixture became faint yellow homogeneously after shaking on an oscillator. Then, 1 mL of deionized water was injected into the centrifuge tube. The faint yellow mixture was dissolved in deionized water under ultrasound treatment for 3 h, forming a brown solution. The solution was then added to 1 g of γ-Al_2_O_3_ powder. The mixture was shaken on an oscillator and treated under ultrasound treatment for another 1 h. After impregnation, the brown slurry was dried at 100 °C for 12 h and then calcinated at 600 °C in a muffle furnace for 6 h.

#### Synthesis of **MoAl**

MoO_3_/Al_2_O_3_ catalyst was defined as MoAl. MoAl was also synthesized by the facile impregnation method. Typically, 0.1635 g of (NH_4_)_6_Mo_7_O_24_·4H_2_O (precursor of MoO_3_) and 2 mL of deionized water were loaded in a 10-mL disposable centrifuge tube. (NH_4_)_6_Mo_7_O_24_·4H_2_O was dissolved in deionized water under ultrasound treatment for 1 h, forming a colorless solution. The solution was then added to 1 g of γ-Al_2_O_3_ powder. The mixture was shaken on an oscillator and treated under ultrasound treatment for another 1 h. After impregnation, the brown slurry was dried at 100 °C for 12 h and then calcinated at 600 °C in a muffle furnace for 6 h.

### Characterization

X-ray diffraction (XRD) patterns were carried out on a Bruker D8 Focus operated at 40 kV and 40 mA equipped with graphite filtered Cu K*α* radiation (*λ* = 1.5406 Å) with 2*θ* values from 20° to 70°.

Raman spectroscopy was performed on a Renishaw inVia reflex Raman spectrometer using a 532 nm Ar ion laser beam under ambient conditions.

Scanning electron microscopy (SEM) was characterized on Hitachi S-4800 under 5 kV. Transmission electron microscopy (TEM) measurements were performed on a JEM-2100F transmission electron microscope under 100 kV. AC-HAADF-STEM measurements were carried out on a JEM ARM 200F from JEOL operated at 200 kV.

The X-ray absorption spectra (XAS) data at Mo and Fe K-edge were collected at BL14W1 station in Shanghai Synchrotron Radiation Facility (SSRF). The electron storage ring was operated at 3.5 GeV with a maximum current of 250 mA. The data were collected at room temperature in a fluorescence excitation mode. All samples were pelletized as disks of 13 mm diameter with 1 mm thickness.

X-ray photoelectron spectroscopy (XPS) was recorded on a PerkinElmer PHI 1600 ESCA instrument with an Al K*α* X-ray source (*hν* = 1486.6 eV). The binding energies were calibrated by the C 1s peak at 284.5 eV.

O_2_ temperature-programmed oxidation (O_2_-TPO) was performed on the Micromeritics Auto Chem II 2920 chemisorption apparatus. Typically, 100 mg of the spent catalysts were heated in a U-type reactor at 300 °C for 1 h under Ar flow and then cooled to 100 °C. The pretreated samples were heated to 700 °C at a rate of 10 °C/min in a mixture of 10 vol% O_2_ in He (30 mL/min)

NH_3_ temperature-programmed desorption (NH_3_-TPD) was also performed on the Micromeritics Auto Chem II 2920 chemisorption apparatus. Typically, 100 mg of the redox catalysts were heated in a U-type reactor at 300 °C for 1 h under Ar flow and then cooled to 100 °C. NH_3_ was adsorbed using a flow of 5 vol% NH_3_ in He (20 mL/min) for 1 h. Finally, the samples were heated to 700 °C at a rate of 10 °C/min under the flow of He (30 mL/min).

H_2_ temperature-programmed reduction (H_2_-TPR) was also performed on the Micromeritics Auto Chem II 2920 chemisorption apparatus. Typically, 100 mg of the redox catalysts were heated in a U-type reactor at 300 °C for 1 h under Ar flow and then cooled to 80 °C. Finally, the samples were heated to 800 °C at a rate of 10 °C/min in a mixture of 10 vol% H_2_ in Ar (30 mL/min).

C_3_H_8_ temperature-programmed reduction (C_3_H_8_-TPR) was measured on the same apparatus, and the output gas products were analyzed on Hiden QIC-20 mass spectrometer (C_3_H_8_, C_3_H_6_, CO_2_, CH_4_, H_2_, and H_2_O, m/e equals to 29, 41, 44, 16, 2 and 18, respectively). Typically, 100 mg of the redox catalysts were heated in a U-type reactor at 300 °C for 1 h under Ar flow and cooled to 80 °C. The pretreated samples were then heated to 600 °C at a rate of 10 °C/min in a mixture of 15 vol% C_3_H_8_ in He (10 mL/min).

C_3_H_8_-D_2_ temperature-programmed surface reduction (C_3_H_8_-D_2_-TPSR) was measured on the same apparatus, and the output gas products were analyzed on Hiden QIC-20 mass spectrometer (C_3_H_8_, C_3_H_7_D, D_2_, H_2_, and HD, m/e equals to 29, 30, 4, 2 and 3, respectively). Typically, 100 mg of the redox catalysts were heated in a U-type reactor at 300 °C for 1 h under Ar flow and cooled to 50 °C. The pretreated samples were heated to 600 °C at a rate of 10 °C/min in a mixture of 5 vol% C_3_H_8_, 5 vol% D_2_ in He (10 mL/min).

C_3_H_8_ isothermal and pulse experiments were also carried out on the same apparatus, and the output products were also measured on Hiden QIC-20 mass spectrometer (C_3_H_8_, C_3_H_6_, CO_2_, CH_4_, H_2_, and H_2_O, m/e equals to 29, 41, 44, 16, 2 and 18, respectively). Typically, 100 mg samples were pretreated in a U-type reactor at 570 °C for 30 minutes under Ar flow. C_3_H_8_ was then injected into the reactor for 1 h. The exhausted gas was analyzed by an online mass spectrometer.

In situ diffuse reflectance infrared Fourier transform spectroscopy (in situ DRIFTS) was conducted on a Thermo Scientific Nicolet IS50 spectrometer equipped with a Harrick Scientific DRIFT cell and a mercury-cadmium-telluride MCT detector cooled by liquid N_2_.

C_3_H_8_-DRIFTS. The redox catalysts were heated at 570 °C for 30 minutes under Ar flow and then cooled to 50 °C. Background spectra were recorded at the desired temperature. Sample spectra were collected every 50 °C from 50 to 570 °C. The pretreated samples were kept in a mixture of 15 vol% C_3_H_8_ in He (10 mL/min) for 4 minutes at all temperatures. Background spectra were subtracted simultaneously to obtain the desired IR spectra.

N_2_-DRIFTS. To verify the consecutively out-diffusion of lattice oxygen, the spent 1Mo9FeAl redox catalyst was heated under N_2_ flow at the reaction temperature. The spent 1Mo9FeAl redox catalyst experienced the C_3_H_8_-DRIFTS experiment was quickly cooled to 50 °C and the spectrum was recorded. The sample was then kept under N_2_ (20 mL/min) flow for another 1 h and warmed to 570 °C. The spectrum was collected after being heated under N_2_ at 570 °C for 10 minutes. The sample was then quickly cooled to 50 °C, and the spectrum was also recorded.

NH_3_-DRIFTS. Catalysts to be measured were pretreated at 400 °C for 30 minutes under Ar flow to remove the absorbed water and gas and cooled to 50 °C. Background spectra were then recorded. A mixture of 10 vol% NH_3_ in He (20 mL/min) was introduced, and sample spectra were collected every minute. Background spectra were subtracted to obtain the desired IR spectra.

Thermal gravity analyses were operated on Setaram Themys thermogravimetry apparatus. 30 mg of the redox catalysts were heated in the suspended crucible at 570 °C for 30 minutes under Ar flow. A mixture of 15 vol% C_3_H_8_ or C_3_H_6_ in He (50 mL/min) was then introduced, and the TG profiles were recorded at 570 °C for 1 h.

In situ Raman spectroscopy was performed on a Horiba LabRAM HR Evolution Raman spectrometer using a 532 nm Ar ion laser beam. Raman spectra were recorded from 50 to 570 °C every 50 °C in the flow of 15 vol% C_3_H_8_ in He (20 mL/min) after pretreated at 300 °C for 2 h to remove the influence of hydrated species.

In situ XRD experiments were operated on a Rigaku Smartlab operated at 60 kV and 220 mA equipped with graphite filtered Cu K*α* radiation (*λ* = 1.5406 Å) with 2*θ* values from 20° to 70° at a scan speed of 10 °/min. XRD patterns were collected from 50 to 570 °C every 50 °C in the flow of 15 vol% C_3_H_8_ in He (20 mL/min) after being pretreated at 300 °C for 2 h.

### Kinetics experiments

Reduction kinetics experiments of the as-prepared redox catalysts were operated on Setaram Themys thermogravimetry apparatus for quantifying the weight change of the redox catalysts. Typically, 20 mg of the redox catalysts with an average radius of 200 μm was heated at 570 °C under the flow of 2 vol% C_3_H_8_ in N_2_ for 90 minutes. To remove the external and internal mass transfer limitation, the kinetics experiments were operated at high gas flow rates (100 mL/min), and the detailed calculation can be found in Supplementary Table 13.

To eliminate the buoyancy and drag effects, the reduction kinetics experiments were conducted with a blank experiment using the blank crucible at the same reaction conditions. The solid conversion *X* was calculated using the corrected sample weight based on Eq. 1.

1 $$X=\frac{{m}_{0}-{m}_{t}}{{m}_{0}-{m}_{\infty }}.$$

Where the *m*_*0*_ represents the weight of the fresh sample, *m*_*t*_ is the weight at time *t* during the reduction, ${m}_{\infty }$ is the ultimate mass of the sample at the end of the reduction; bulk diffusion coefficient, *D*, and oxygen surface exchange coefficient, *k*.

External and internal mass transfer limitations were eliminated by increasing the gas flow rates based on the Mears criterion and the Weisz–Prater criterion.

The external mass transfer limitation was excluded if the Mears number calculated from Eq. 2 is less than 0.15.

2 $${{{{{\rm{MR}}}}}}=\frac{-{{{{{{\rm{r}}}}}}}^{{\prime} }_{{{{{{{\rm{C}}}}}}}_{3}{{{{{{\rm{H}}}}}}}_{8}{{{{{\rm{obs}}}}}}}\left(1-{{{{{\rm{\phi }}}}}}\right){{{{{\rm{\rho }}}}}}_{1{{{{{\rm{Mo}}}}}}9{{{{{\rm{FeAl}}}}}}}\left(\frac{{{D}}}{2}\right)^{{n}}}{{{{{{k}}}}}_{{{{{{\rm{c}}}}}}}{{{{{{\rm{C}}}}}}}_{{{{{{{\rm{C}}}}}}_{3}}{{{{{{\rm{H}}}}}}}_{8}{{{{{\rm{b}}}}}}}}.$$

where $-{{{{{{\rm{r}}}}}}}^{{\prime} }_{{{{{{{\rm{C}}}}}}}_{3}{{{{{{\rm{H}}}}}}}_{8}{{{{{\rm{obs}}}}}}}$ (mol/g-cat s) is the observed reduction rate under C_3_H_8_-N_2_ flow, ${{{{{{\rm{\rho }}}}}}}_{1{{\mbox{Mo}}}9{{\mbox{FeAl}}}}$ is the solid density of 1Mo9FeAl, ${{{{{\rm{\phi }}}}}}$ is the porosity of 1Mo9FeAl, *D* is the diameter of 1Mo9FeAl particle, *n* is the reaction order of C_3_H_8_, *k*_c_ is the mass transfer coefficient of C_3_H_8_ in N_2_, and ${{{\mbox{C}}}}_{{{{{\mbox{C}}}}_{3}{{\mbox{H}}}}_{8}{{\mbox{b}}}}$ is the bulk concentration of C_3_H_8_.

The internal mass transfer limitation was eliminated if the Weisz–Prater parameter calculated from Eq. 3 is significantly less than 1.

3 $${{{{{{\rm{C}}}}}}}_{{{{{{\rm{WP}}}}}}}=\frac{-{{{{{{\rm{r}}}}}}}^{{\prime} }_{{{{{{{\rm{C}}}}}}}_{3}{{{{{{\rm{H}}}}}}}_{8}{{{{{\rm{obs}}}}}}}{{{{{\rm{\rho }}}}}}_{1{{{{{\rm{Mo}}}}}}9{{{{{\rm{FeAl}}}}}}}{\left(\frac{{{D}}}{2}\right)}^{2}}{{{{D}}}_{{{{{{\rm{eff}}}}}}}{{{{{{\rm{C}}}}}}}_{{{{{{{\rm{C}}}}}}}_{3}{{{{{{\rm{H}}}}}}}_{8}{{{{{\rm{s}}}}}}}}.$$

where *D*_*eff*_ is the effective diffusivity of C_3_H_8_ in N_2_ and ${{{\mbox{C}}}}_{{{{{\mbox{C}}}}_{3}{{\mbox{H}}}}_{8}{{\mbox{s}}}}$ is the concentration of C_3_H_8_ at the surface of 1Mo9FeAl particle.

Supplementary Table 13 presented the detailed parameters involved in the calculation for MR and ${{{\mbox{C}}}}_{{{\mbox{WP}}}}$ in determining the external and internal mass transfer limitation. Based on the calculation results, both external and internal mass transfer limitation was eliminated in this work.

### Redox test

Redox tests were carried out in a fixed-bed reactor equipped with a quartz tube (8 mm inner diameter and 24 cm length) at 0.14 MPa. Typically, 0.5 g of as-prepared 1MoxFeAl redox catalysts (20–40 mesh) diluted with 1 mL of quartz particles was loaded in the quartz tubular reactor. Prior to the reduction period, the sample was heated to 570 °C under flowing nitrogen (17 mL/min). Afterward, 1MoxFeAl was reduced in the mixture of propane (4 mL/min) and nitrogen (17 mL/min). The Gas Hour Space Velocity (GHSV) was about 3000 h^−1^. During the switching between propane and air flows (switch over between the reduction and regeneration period), an inert period (nitrogen only, 17 mL/min) of about 10 minutes was essential to avoid direct contact between propane and air. After heating in air for regeneration at 570 °C, one redox cycle was completed. The time for reduction, regeneration, and inert period was set to be 4, 10, and 10 minutes, respectively. The stability test was performed over 1Mo9FeAl for 300 redox cycles.

The outlet gas was analyzed by an online GC (2060) equipped with a flame ionization detector (Chromosorb 102 column) and a thermal conductivity detector (Al_2_O_3_ Plot column) and GC (Micro GC 490, Agilent) equipped with three channels (MS 5A Plot column, Pora Plot Q, and PoraPlot Q). The instantaneous propane conversion and gas phase products selectivity was determined from Eq. 4 and Eq. 5

4 $${{{{{\rm{Con}}}}}}\,(\%)=100*\frac{{[{{{\mbox{F}}}}_{{{{\mbox{C}}}}_{3}{{{\mbox{H}}}}_{8}}]}_{{{\mbox{inlet}}}}-{[{{{\mbox{F}}}}_{{{{\mbox{C}}}}_{3}{{{\mbox{H}}}}_{8}}]}_{{{\mbox{outlet}}}}}{{[{{{\mbox{F}}}}_{{{{\mbox{C}}}}_{3}{{{\mbox{H}}}}_{8}}]}_{{{\mbox{inlet}}}}}.$$

5 $${{{{{\rm{Sel}}}}}}\,(\%)=100*{{{{{\rm{n}}}}}}_{{{{{\rm{i}}}}}}*\frac{{[{{{\mbox{F}}}}_{{{\mbox{i}}}}]}_{{{\mbox{outlet}}}}}{{{\sum {{{\mbox{n}}}}_{{{\mbox{i}}}}}*[{{\mbox{F}}}_{{{\mbox{i}}}}]}_{{{\mbox{outlet}}}}}.$$

The total selectivity of product i based on all products was calculated from Eq. 6.

6 $${{{{{\rm{Sel}}}}}}\,\left(\%\right)=100*{{{{{\rm{n}}}}}}_{{{{{\rm{i}}}}}}*\frac{{[{{{\mbox{F}}}}_{{{\mbox{i}}}}]}_{{{\mbox{outlet}}}}}{{[{{{\mbox{F}}}}_{{{{\mbox{C}}}}_{3}{{{\mbox{H}}}}_{8}}]}_{{{\mbox{inlet}}}}-{[{{{\mbox{F}}}}_{{{{\mbox{C}}}}_{3}{{{\mbox{H}}}}_{8}}]}_{{{\mbox{outlet}}}}}.$$

The instantaneous propylene productivity was calculated on the basis of Eq. 7, and the accumulative yield of propylene within 10 minutes during the dehydrogenation stage was determined from Eq. 8.

7 $${{{{{\rm{Productivity}}}}}}=\frac{{[{{{\mbox{F}}}}_{{{{\mbox{C}}}}_{3}{{{\mbox{H}}}}_{6}}]}_{{{\mbox{outlet}}}}}{m}.$$

8 $${{{{{\rm{Yield}}}}}}=\int {{\mbox{Productivity}}}\,{{\mbox{d}}}{t}.$$

The accumulative conversion (Eq. 9) and selectivity (Eq. 10) within *t* minutes were defined as the integral conversion and selectivity in Eqs. 2 and 3 dividing *t* minutes during the dehydrogenation stage.

9 $${{{{{{\rm{Con}}}}}}}_{{{{{{\rm{int}}}}}}}\,\left(\%\right)=\frac{\int {{\mbox{Con}}}\,\left(\%\right)\,{{\mbox{d}}}t}{t}.$$

10 $${{{{{{\rm{Sel}}}}}}}_{{{{{{\rm{int}}}}}}}\,\left(\%\right)=\frac{\int {{\mbox{Sel}}}\,\left(\%\right)\,{{\mbox{d}}}t}{t}.$$

i = component i in exhausted gases; n_i_ = number of carbon atoms of component i; ${[{{{\mbox{F}}}}_{{{\mbox{i}}}}]}_{{{\mbox{outlet}}}}$ = molar flow rate of component i out of reactor (mol/h); ${[{{{\mbox{F}}}}_{{{{\mbox{C}}}}_{3}{{{\mbox{H}}}}_{6}}]}_{{{\mbox{outlet}}}}$ = molar flow of propylene out of reactor (mol/h); ${[{{{\mbox{F}}}}_{{{{\mbox{C}}}}_{3}{{{\mbox{H}}}}_{8}}]}_{{{\mbox{outlet}}}}$ = molar flow of propane out of reactor (mol/h); ${[{{{\mbox{F}}}}_{{{{\mbox{C}}}}_{3}{{{\mbox{H}}}}_{8}}]}_{{{\mbox{inlet}}}}$ = molar flow of propane in of reactor (mol/h); *t* = the time during the dehydrogenation stage (minute); *m* = weight of the redox catalysts.

Based on the internal standard experiments, trace coking or tar was formed under the reduction stage, taking the carbon balance into consideration. To calculate the propylene yields, a carbon mass balance was adopted to calculate the conversion and selectivity.

## Supplementary information

Supplementary Information

## Data Availability

The data generated in this study are provided in Supplementary Information and Source Data file. Source data are provided with this paper.

## Source data

Source Data
