# Supplementary material for: Coupling acid catalysis and selective oxidation over MoO3-Fe2O3 for chemical looping oxidative dehydrogenation of propane
Source: Nat Commun. 2023 Apr 11;14:2039. doi: 10.1038/s41467-023-37818-w (PMC10090184; doi:10.1038/s41467-023-37818-w)
Supplement: Supplementary file 1 — Supplementary Information [file 41467_2023_37818_MOESM1_ESM.pdf]

Supplementary Information

Coupling Acid Catalysis and Selective Oxidation over MoO_3-Fe_2O_3 for Chemical Looping Oxidative Dehydrogenation of Propane

Xianhui Wang,[1,2,3,§] Chunlei Pei,[1,2,3,§] Zhi-Jian Zhao,[1,2,3,§] Sai Chen,[1,2,3,4] Xinyu Li,[1,2,3] Jiachen Sun,[1,2,3] Hongbo Song,[1,2,3] Guodong Sun,[1,2,3,4] Wei Wang,[1,2,3,4] Xin Chang,[1,2,3] Xianhua Zhang,[1,2,3,4] and Jinlong Gong[1,2,3,4]*

[1]*School of Chemical Engineering & Technology, Key Laboratory for Green Chemical Technology of Ministry of Education, Tianjin University, Tianjin 300072, China.*

[2]*Collaborative Innovation Center for Chemical Science & Engineering (Tianjin), Tianjin 300072, China.*

[3]*Haihe Laboratory of Sustainable Chemical Transformations, Tianjin 300192, China.*

[4]*Joint School of National University of Singapore and Tianjin University, International Campus of Tianjin University, Binhai New City, Fuzhou 350207, China.*

[§] These authors contributed equally to this work.

*E-mail: jlgong@tju.edu.cn

Contents

1. Supplementary Figures and Tables

Page S1: Fig. S1. Determination on reaction conditions.

Page S2: Fig. S2. Products distribution over 1Mo9FeAl.

Page S3: Fig. S3. Catalytic performance and fitted curve over 1Mo9FeAl.

Page S4: Fig. S4. The integral conversion and selectivity over 1Mo9FeAl.

Page S5: Fig. S5. O_2-TPO profiles of spent MoO_3-Fe_2O_3 redox catalysts.

Page S6: Fig. S6. Products distribution over 1Mo9FeAl and 1Mo9FeAl-red.

Page S7: Fig. S7. Catalytic performance over regenerated 1Mo9FeAl.

Page S8: Fig. S8. XRD patterns of regenerated 1Mo9FeAl.

Page S9: Fig. S9. XRD patterns cycled 1Mo9FeAl.

Page S10: Fig. S10. XPS measurements of cycled 1Mo9FeAl.

Page S11: Fig. S11. XRD patterns of 1Mo9FeAl for phase transformation.

Page S12: Fig. S12. Pore size distributions of MoO_3-Fe_2O_3 redox catalysts.

Page S13: Fig. S13. SEM images of MoO_3-Fe_2O_3 redox catalysts.

Page S14: Fig. S14. line profile of 1Mo9FeAl.

Page S15: Fig. S15. EXAFS spectra in k space and R space.

Page S16: Fig. S16. EDS line scan of 1Mo9FeAl.

Page S17: Fig. S17. AC-HAADF-STEM image of 1Mo6FeAl.

Page S18: Fig. S18. Shift in diffraction angle for MoO_3-Fe_2O_3 redox catalysts.

Page S19: Fig. S19. XRD pattern of 1Mo3FeAl.

Page S20: Fig. S20. XRD pattern of MoAl.

Page S21: Fig. S21. Raman Spectrum of 1Mo3FeAl.

Page S22: Fig. S22. Raman spectra for MoO_3-Fe_2O_3 redox catalysts.

Page S23: Fig. S23. XPS spectra of Fe 2*p* for MoO_3-Fe_2O_3 redox catalysts.

Page S24: Fig. S24. Fe K-edge XANES spectra of MoO_3-Fe_2O_3 redox catalysts.

Page S25: Fig. S25. XPS measurements of Mo 3*d* for MoO_3-Fe_2O_3 redox catalysts.

Page S26: Fig. S26. NH_3-DRIFTS spectra of MoO_3-Fe_2O_3 redox catalysts.

Page S27: Fig. S27. NH_3-TPD profiles of 1Mo9FeAl at different reaction times.

Page S28: Fig. S28. XPS spectra of Mo 3*d* for spent MoO_3-Fe_2O_3 redox catalysts.

Page S29: Fig. S29. XPS spectra of Fe 2*p* for spent 1Mo9FeAl.

Page S30: Fig. S30. H_2-TPR profile of MoAl.

Page S31: Fig. S31. *In situ* Raman spectra of MoO_3-Fe_2O_3 redox catalysts.

Page S32: Fig. S32. *In situ* DRIFTS spectra of MoO_3-Fe_2O_3 redox catalysts.

Page S33: Fig. S33. *In situ* DRIFTS spectra of regenerated 1Mo9FeAl.

Page S34: Fig. S34. *In situ* XRD patterns of FeAl.

Page S35: Fig. S35. Isothermal *in situ* XRD patterns of MoO_3-Fe_2O_3 redox catalysts.

Page S36: Fig. S36. TG and DTG profiles of MoO_3-Fe_2O_3 redox catalysts.

Page S37: Fig. S37. Products distribution over MoO_3-Fe_2O_3 redox catalysts.

Page S38: Fig. S38. Isothermal reduction of MoO_3-Fe_2O_3 redox catalysts.

Page S39: Fig. S39. H_2O/H_2 ratio for MoO_3-Fe_2O_3 redox catalysts.

Page S40: Table S1. Catalytic performance of reported Fe-based catalysts.

Page S41: Table S2. Catalytic performance of reported ODH catalysts.

Page S42: Table S3. Textural properties of cycled 1Mo9FeAl.

Page S43: Table S4. XPS derived surface composition for cycled 1Mo9FeAl.

Page S44: Table S5. Physical absorption results for MoO_3-Fe_2O_3 redox catalysts.

Page S45: Table S6. EXAFS fitting parameters at Mo K-edge.

Page S46: Table S7. Textural properties of MoO_3-Fe_2O_3 redox catalysts.

Page S47: Table S8. XPS derived surface composition for MoO_3-Fe_2O_3 redox catalysts.

Page S48: Table S9. Fitted NH_3-TPD results for MoO_3-Fe_2O_3 redox catalysts.

Page S49: Table S10. XPS derived Mo^{4+} percentage for spent 1Mo9FeAl.

Page S20: Table S11. XPS derived Mo^{4+} percentage for spent MoAl.

Page S51: Table S12. H_2-TPR data of MoO_3-Fe_2O_3 redox catalysts.

Page S52: Table S13. Elimination of internal and external mass transfer limitation.

Page S53: Table S14. Kinetics coefficients for the reduction of FeAl and 1Mo9FeAl

2. Supplementary References

Supplemental Figures and Tables

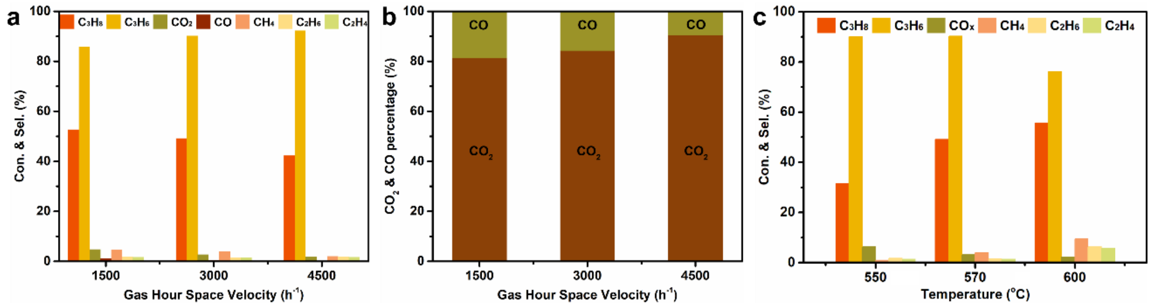

Supplementary Fig. 1. Determination on reaction conditions. (a) Products distribution and (b) CO and CO_2 proportion over 1Mo9FeAl at different GHSV and (c) reaction temperature. Reaction conditions: 0.14 MPa, 0.5 g sample, volumetric C_3H_8/N_2 ratio = 4:17.

The optimal GHSV and reaction temperature are 3000 h^{-1} and 570 °C, respectively.

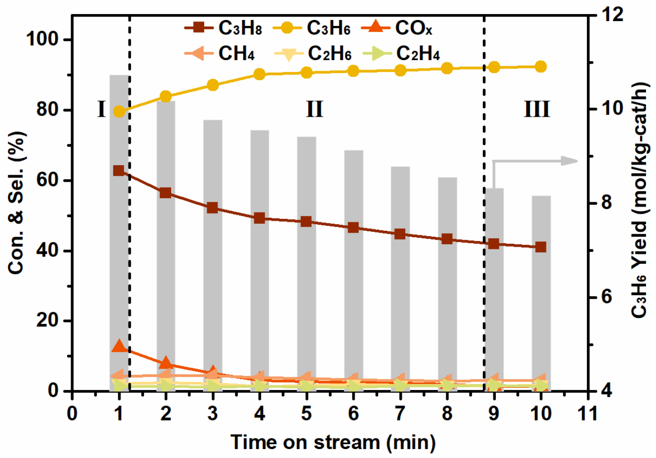

Supplementary Fig. 2. Products distribution over 1Mo9FeAl. Products distribution over 1Mo9FeAl at different reaction time. Reaction conditions: 570 °C, 0.14 MPa, GHSV = 3000 h^{-1}, 0.5 g sample, volumetric C_3H_8/N_2 ratio = 4:17.

The dehydrogenation process was divided into three characteristic stages based on the CO_x (CO and CO_2) content: over-oxidation, oxidative dehydrogenation, and non-oxidative dehydrogenation.[28]

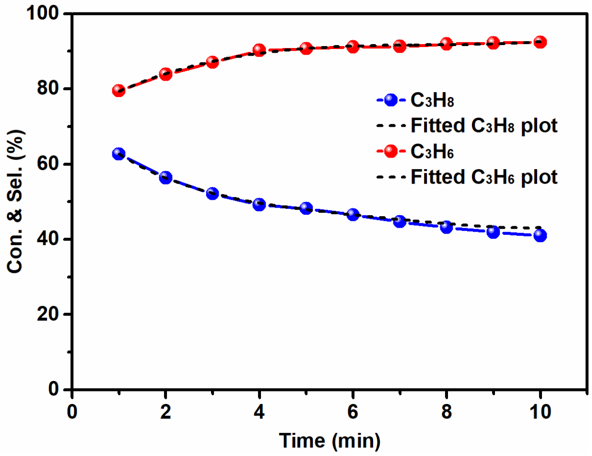

Supplementary Fig. 3. Catalytic performance and fitted curve over 1Mo9FeAl. Catalytic performance of chemical looping oxidative dehydrogenation of propane and fitted curve over 1Mo9FeAl at different reaction time. Reaction conditions: 570 °C, 0.14 MPa, GHSV = 3000 h^{-1}, 0.5 g sample, volumetric C_3H_8/N_2 ratio = 4:17.

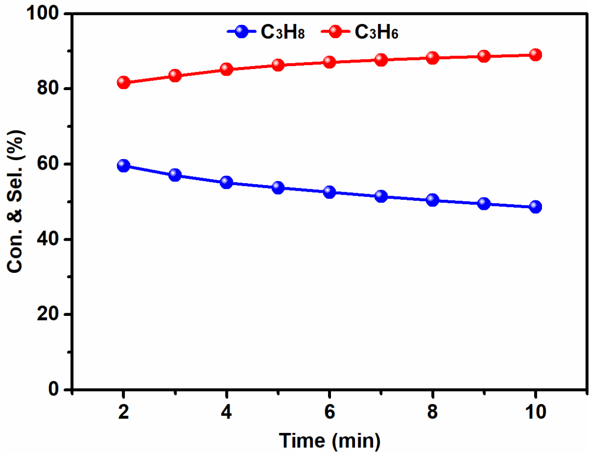

Supplementary Fig. 4. The integral conversion and selectivity over 1Mo9FeAl. The integral conversion and selectivity over 1Mo9FeAl at different reaction time. Reaction conditions: 570 °C, 0.14 MPa, GHSV = 3000 h^{-1}, 0.5 g sample, volumetric C_3H_8/N_2 ratio = 4:17.

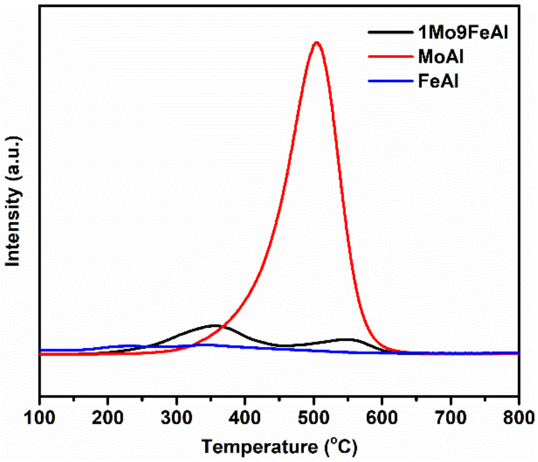

Supplementary Fig. 5. O_2-TPO profiles of spent MoO_3-Fe_2O_3 redox catalysts. O_2-TPO profiles of spent FeAl, 1Mo9FeAl and MoAl, ramping from 100 °C to 700 °C with a rate of 10 °C/min in a mixture of 10 vol% O_2 in He (30 mL/min). The a.u. stands for arbitrary units.

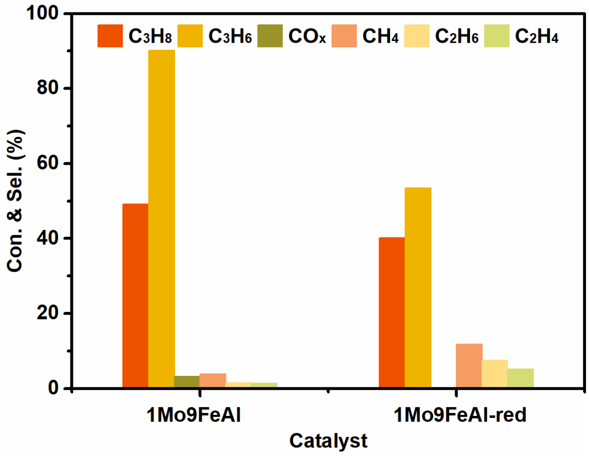

Supplementary Fig. 6. Products distribution over 1Mo9FeAl and 1Mo9FeAl-red. Products distribution of chemical looping oxidative dehydrogenation of propane over 1Mo9FeAl and 1Mo9FeAl-red samples. Reaction conditions: 570 °C, 0.14 MPa, GHSV = 3000 h^{-1}, 0.5 g sample, volumetric C_3H_8/N_2 ratio = 4:17.

After reduced under 10 vol% H_2-N_2 flow at 450 °C for 1 h, lattice oxygen corresponding to γ-Fe_2O_3 to Fe_3O_4 was completely consumed. The reduced redox catalyst was defined as 1Mo9FeAl-red. H_2 was then switched off and 1Mo9FeAl-red was heated to 570 °C in N_2 flow. Finally, the dehydrogenation reaction was carried out over 1Mo9FeAl-red under identical reaction conditions as 1Mo9FeAl.

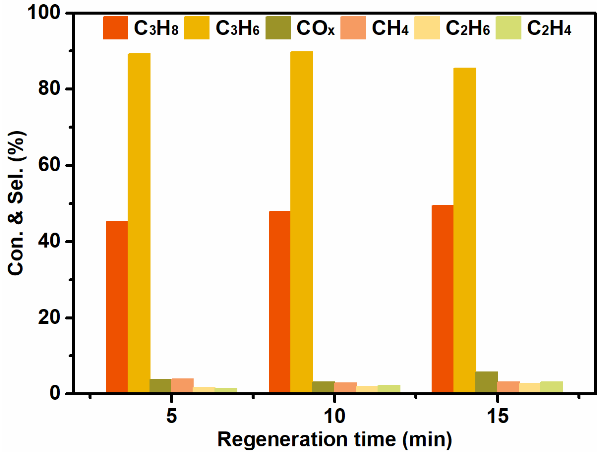

Supplementary Fig. 7. Catalytic performance over regenerated 1Mo9FeAl. Catalytic performance of chemical looping oxidative dehydrogenation of propane over spent 1Mo9FeAl with different regeneration time. Reaction conditions: 570 °C, 0.14 MPa, GHSV = 3000 h^{-1}, 0.5 g sample, volumetric C_3H_8/N_2 ratio = 4:17.

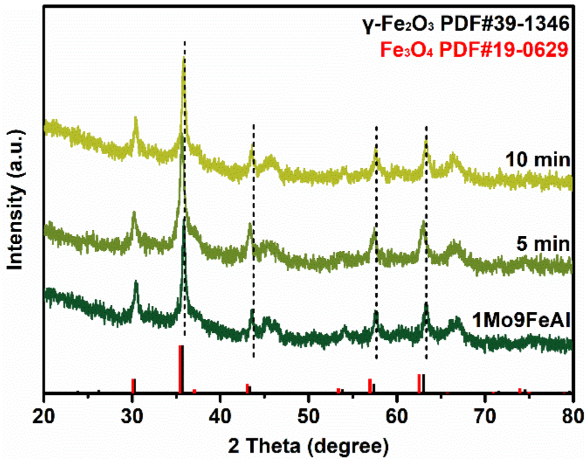

Supplementary Fig. 8. XRD patterns of regenerated 1Mo9FeAl. XRD patterns of regenerated 1Mo9FeAl with different regeneration time. The a.u. stands for arbitrary units.

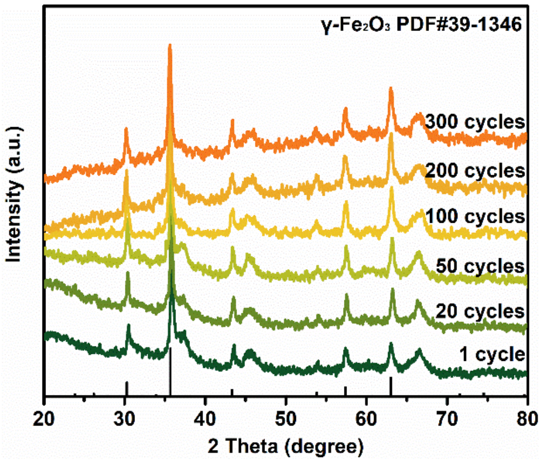

Supplementary Fig. 9. XRD patterns of cycled 1Mo9FeAl. XRD patterns of 1Mo9FeAl undergo different redox cycles. The a.u. stands for arbitrary units.

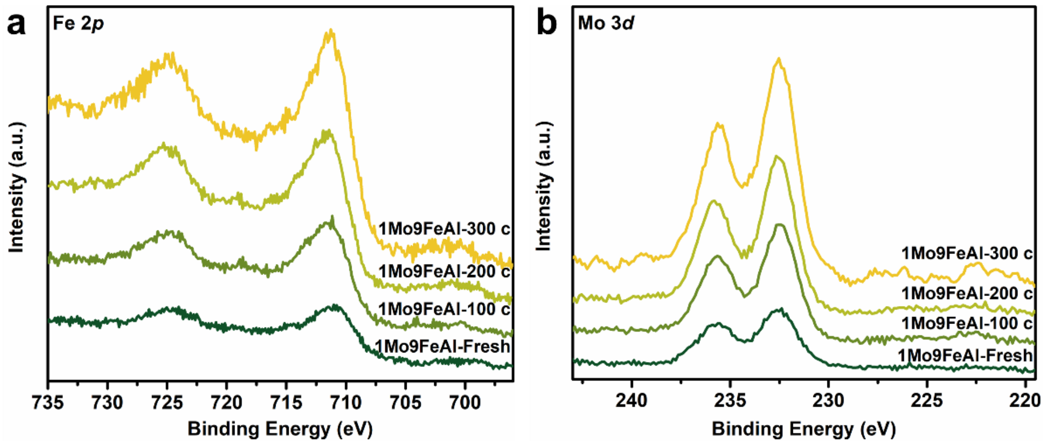

Supplementary Fig. 10. XPS measurements of cycled 1Mo9FeAl. XPS spectra of (a) Fe 2*p*, (b) Mo 3*d* for regenerated 1Mo9FeAl redox catalyst. The a.u. stands for arbitrary units.

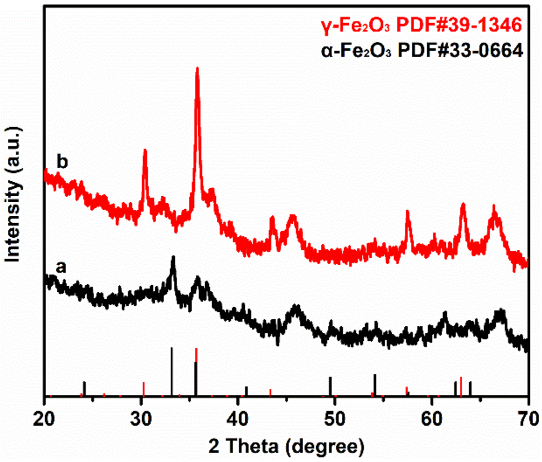

Supplementary Fig 11. XRD patterns of 1Mo9FeAl for phase transformation. XRD patterns of (a) caicined 1Mo9FeAl and (b) as-prepared 1Mo9FeAl. The a.u. stands for arbitrary units.

It is obvious that α-Fe_2O_3 in the redox catalyst was transformed into γ-Fe_2O_3 after one redox cycle based on the XRD patterns. α-Fe_2O_3 in the calcined redox catalysts were reduced to Fe_3O_4 after the oxidative dehydrogenation reaction, and the formed Fe_3O_4 was easily re-oxidized to γ-Fe_2O_3 under air flow in the regeneration reaction.[1,2] However, γ-Fe_2O_3 is metastable and it experienced a phase transformation to α-Fe_2O_3 at high temperature. Molybdenum is known as a refractory material, and it effectively restricted the phase transformation.[3] Consequently, α-Fe_2O_3 in the redox catalysts was transformed into γ-Fe_2O_3 rather than keeping the original α-Fe_2O_3 structure after one redox cycle.

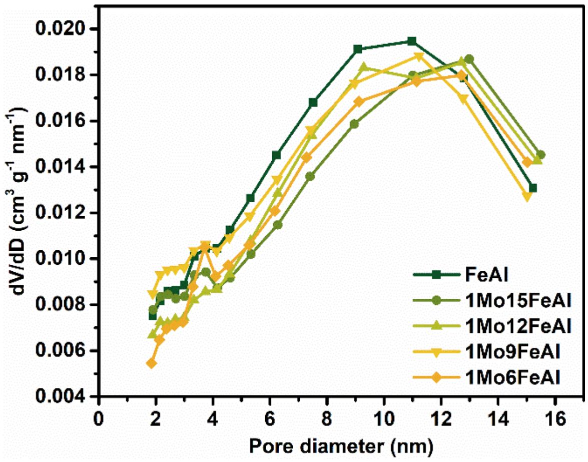

Supplementary Fig. 12. Pore size distribution of MoO_3-Fe_2O_3 redox catalysts. Pore size distributions of as-prepared FeAl and 1MoxFeAl (x = 15, 12, 9, 6).

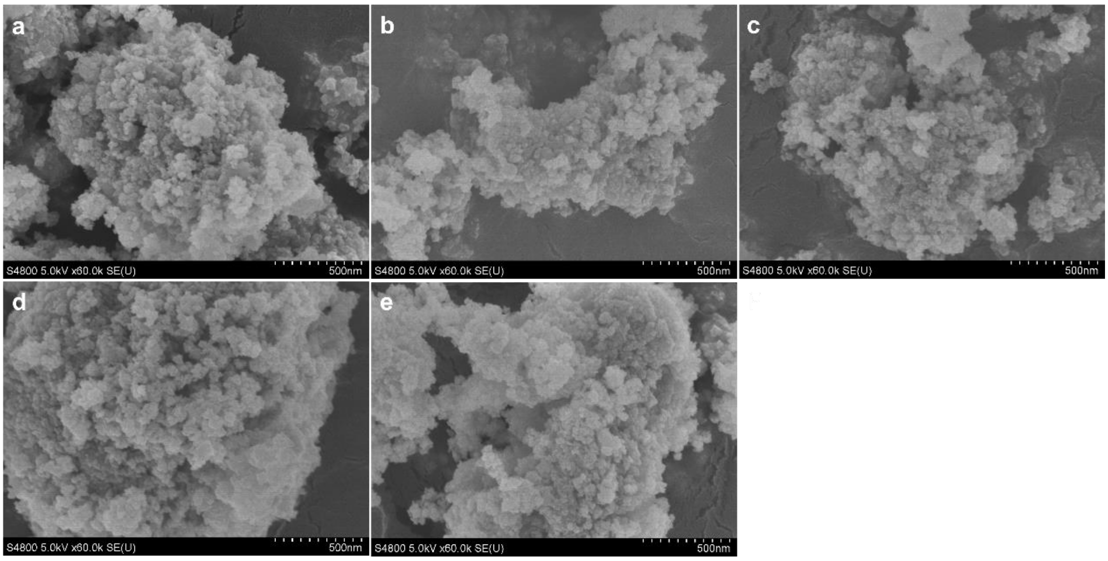

Supplementary Fig. 13. SEM images of MoO_3-Fe_2O_3 redox catalysts. SEM images of as-prepared (a) FeAl, (b) 1Mo15FeAl, (c) 1Mo12FeAl, (d) 1Mo9FeAl, (e) 1Mo6FeAl.

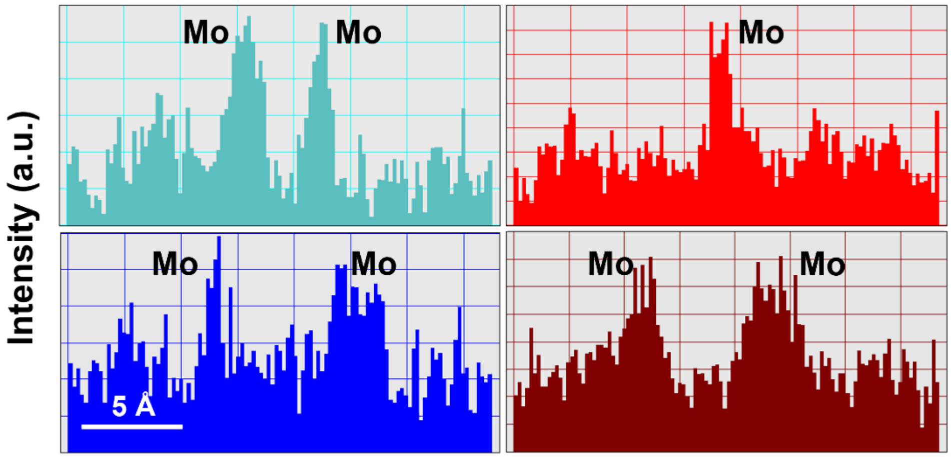

Supplementary Fig. 14. Line profile of 1Mo9FeAl. Corresponding line profile of 1Mo9FeAl in Fig. 2a. The a.u. stands for arbitrary units.

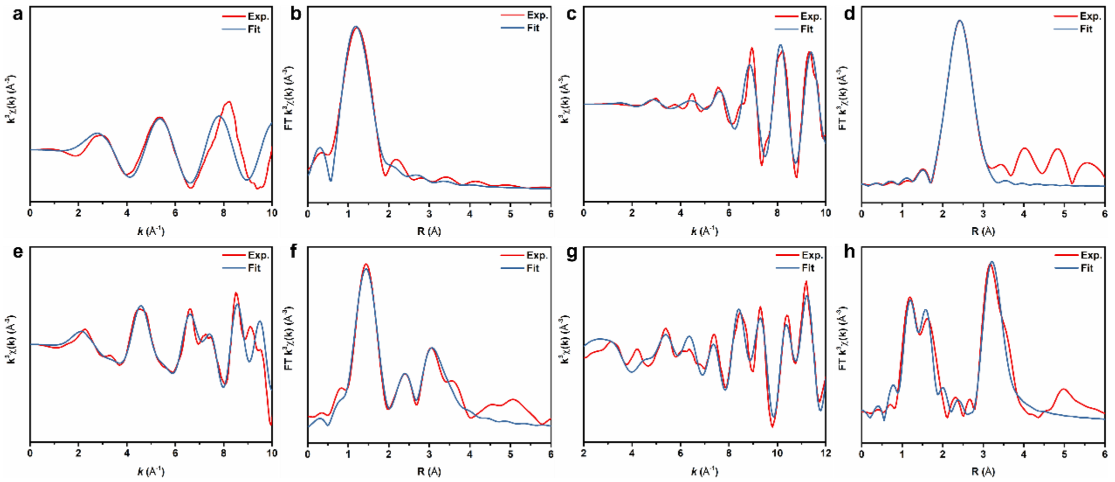

Supplementary Fig. 15. EXAFS spectra in *k* space and *R* space. Fourier-transformed magnitude of EXAFS spectra in *k* space and *R* space. (a, b) 1Mo9FeAl, (c, d) Mo foil, (e, f) MoO_2, and (g, h) MoO_3. The detailed fit parameters are shown in Table S6.

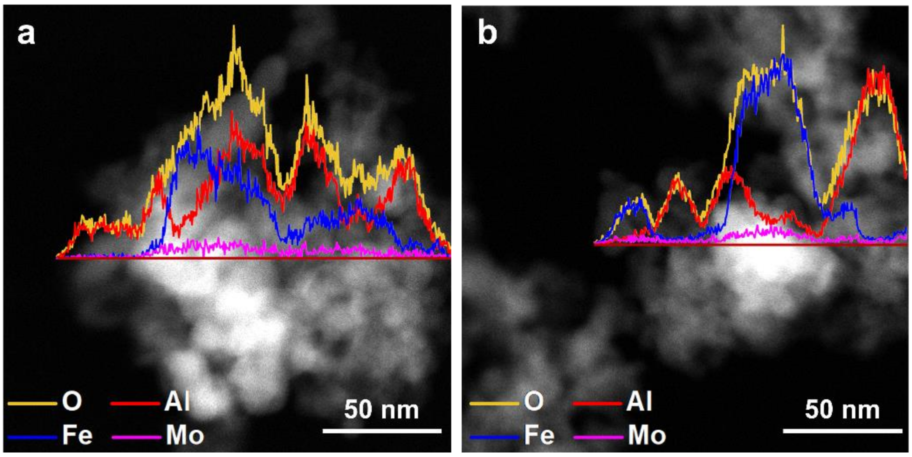

Supplementary Fig. 16. EDS line scan of 1Mo9FeAl. EDS line scan of different regions (a, b) for 1Mo9FeAl.

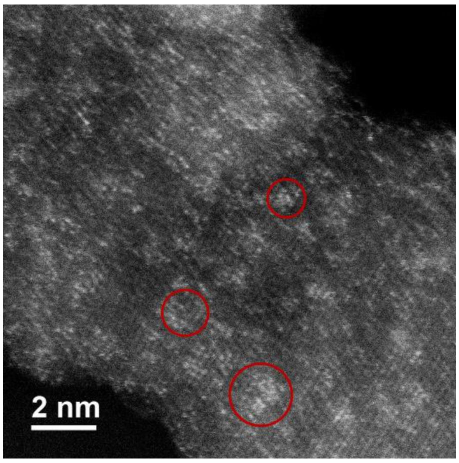

Supplementary Fig. 17. AC-HAADF-STEM image of 1Mo6FeAl.

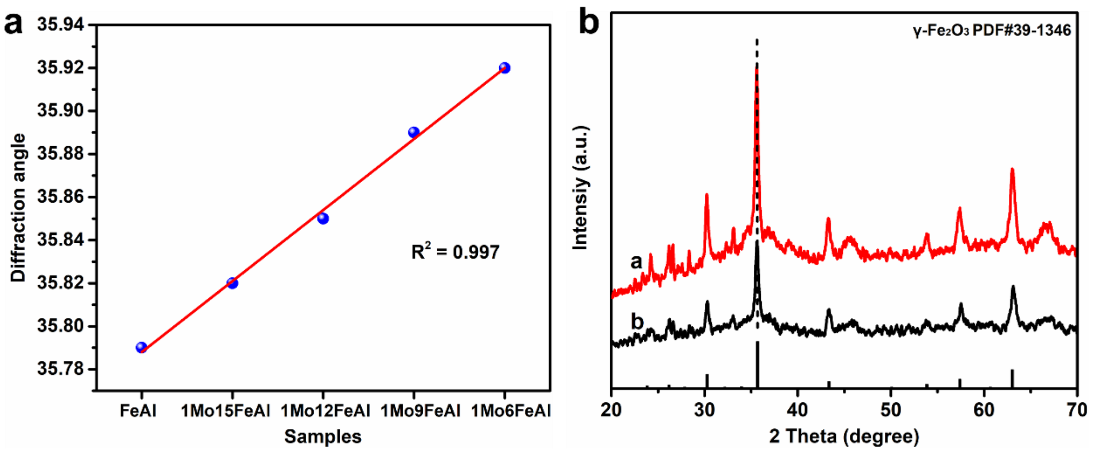

Supplementary Fig. 18. Shift in diffraction angle for MoO_3-Fe_2O_3 redox catalysts. (a) Dependence of diffraction angel for a range of Mo contents for FeAl and 1MoxFeAl (x = 15, 12, 9, 6), (b) XRD pattern of (a) FeAl and (b) the physical mixture of FeAl and MoAl. The a.u. stands for arbitrary units.

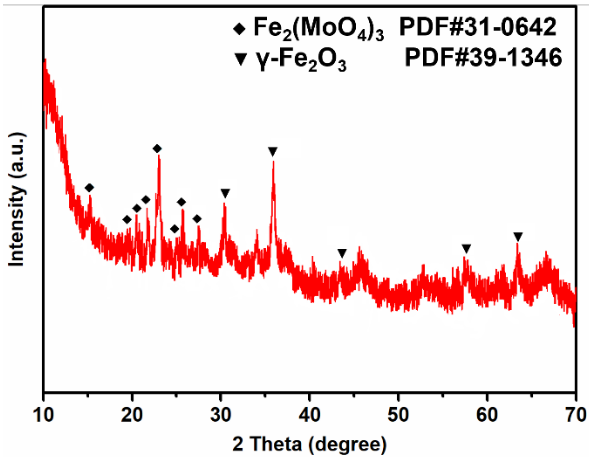

Supplementary Fig. 19. XRD pattern of 1Mo3FeAl. The a.u. stands for arbitrary units.

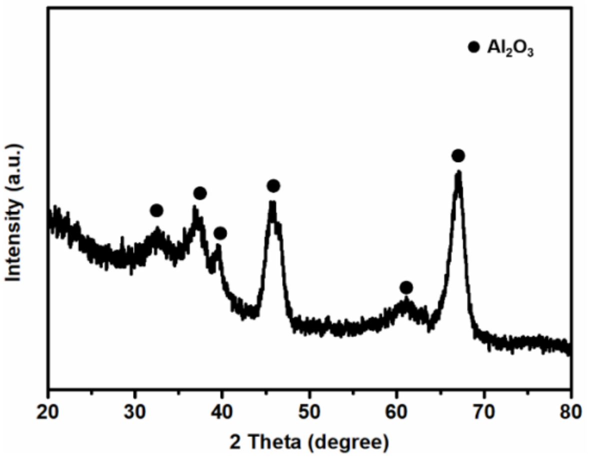

Supplementary Fig. 20. XRD pattern of MoAl. The a.u. stands for arbitrary units.

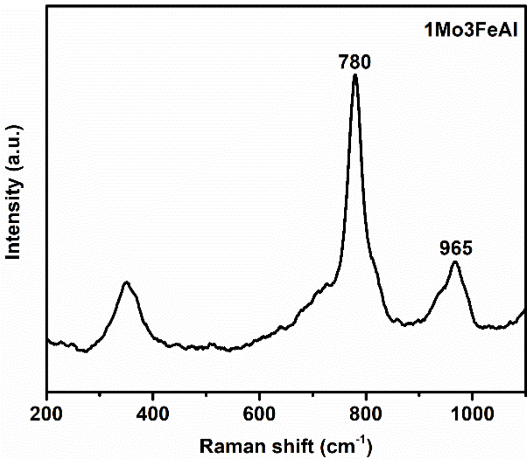

Supplementary Fig. 21. Raman Spectrum of 1Mo3FeAl. The a.u. stands for arbitrary units.

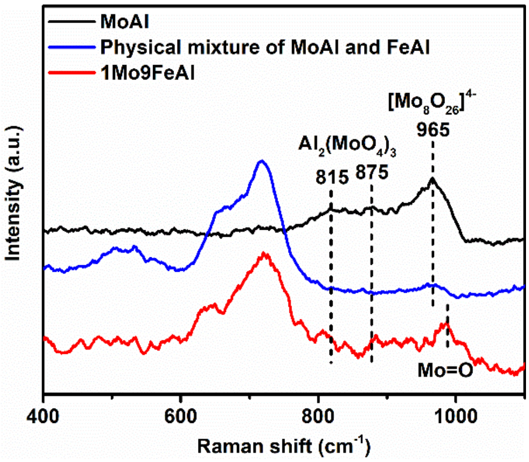

Supplementary Fig. 22. Raman spectra for MoO_3-Fe_2O_3 redox catalysts. Comparison between the Raman spectra of MoAl, 1Mo9FeAl, and the physical mixture of MoAl and FeAl. The a.u. stands for arbitrary units.

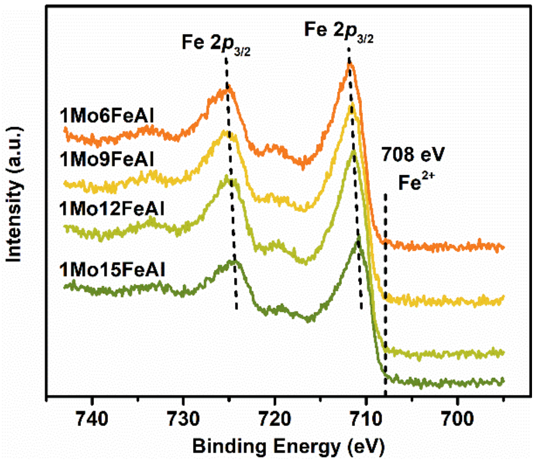

Supplementary Fig. 23. XPS measurements of Fe 2*p* for MoO_3-Fe_2O_3 redox catalysts. XPS spectra of Fe 2*p* for 1Mo15FeAl, 1Mo12FeAl, 1Mo9FeAl, and 1Mo6FeAl on Axis Supra from Kratos Analytical Ltd., UK. The a.u. stands for arbitrary units.

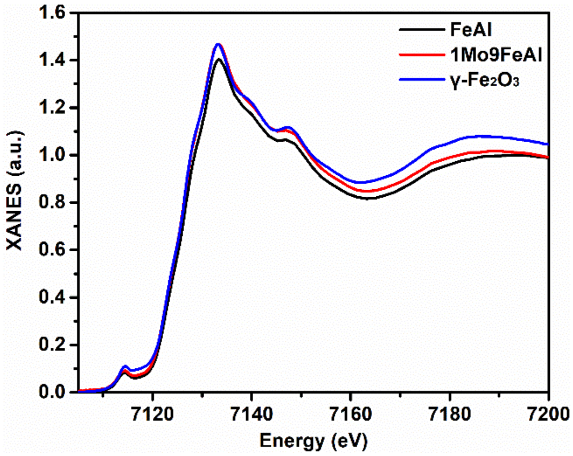

Supplementary Fig. 24. Fe K-edge XANES spectra of MoO_3-Fe_2O_3 redox catalysts. Fe K-edge XANES spectra of FeAl, 1Mo9FeAl, and γ-Fe_2O_3. The a.u. stands for arbitrary units.

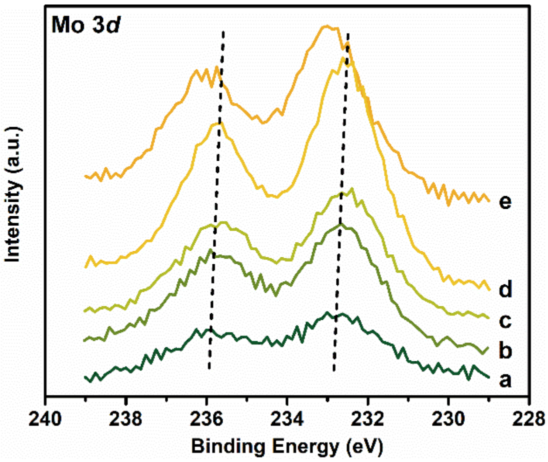

Supplementary Fig. 25. XPS measurements of Mo 3*d* for MoO_3-Fe_2O_3 redox catalysts. XPS spectra of Mo 3*d* for (a) 1Mo15FeAl, (b) 1Mo12FeAl, (c) 1Mo9FeAl, (d) 1Mo6FeAl, and (e) MoAl. The a.u. stands for arbitrary units.

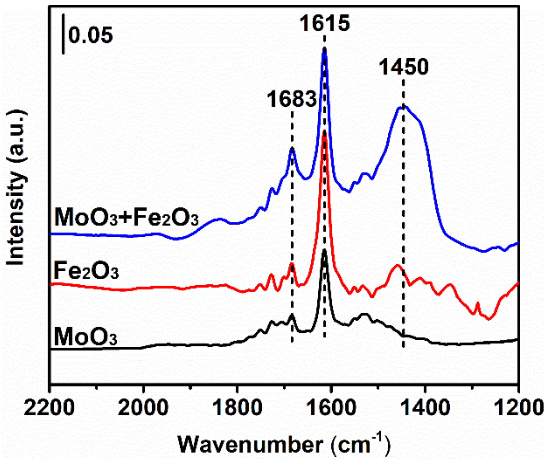

Supplementary Fig. 26. NH_3-DRIFTS spectra of MoO_3-Fe_2O_3 redox catalysts. NH_3-DRIFTS spectra of MoO_3, Fe_2O_3, and the physical mixture of MoO_3 and Fe_2O_3. The a.u. stands for arbitrary units.

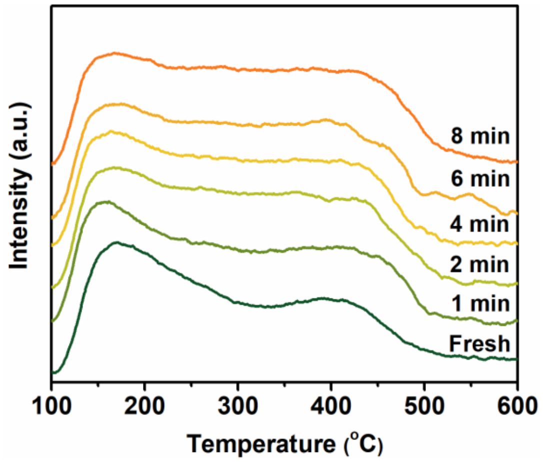

Supplementary Fig. 27. NH_3-TPD profiles of 1Mo9FeAl at different reaction times. NH_3-TPD was performed from 100 °C to 700 °C with a rate of 10 °C/min after being pretreated in a flow of 5 vol% NH_3 in He (20 mL/min) for 1 h at 100 °C The a.u. stands for arbitrary units.

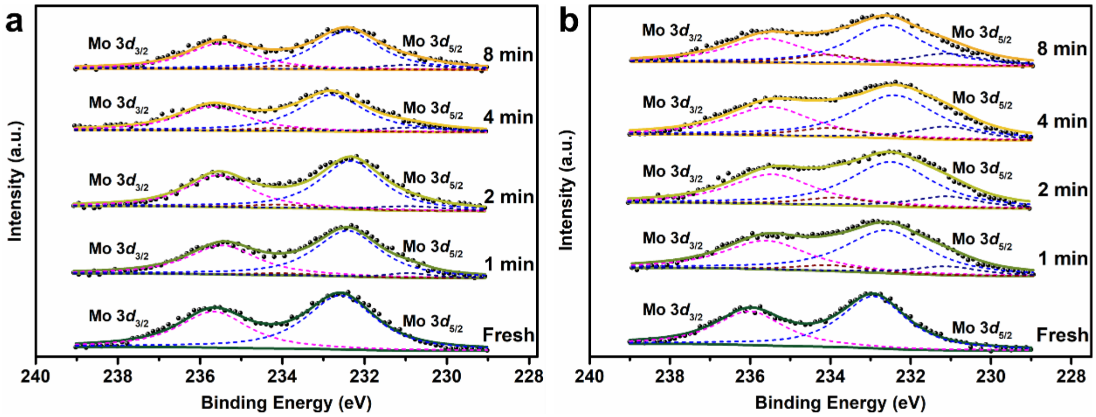

Supplementary Fig. 28. XPS spectra of Mo 3*d* for spent MoO_3-Fe_2O_3 redox catalysts. XPS spectra of Mo 3*d* for (a) 1Mo9FeAl and (b) MoAl at different reaction time. The a.u. stands for arbitrary units.

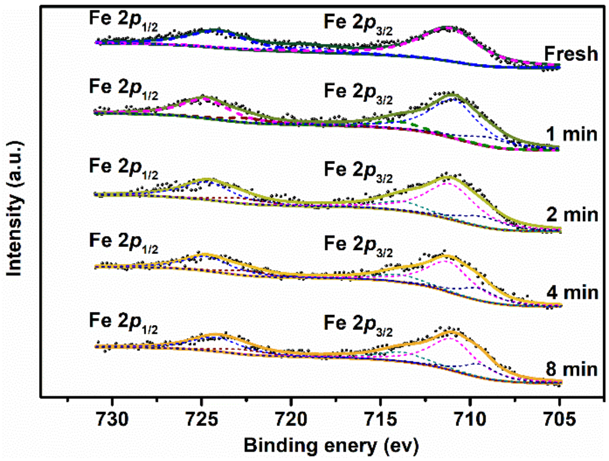

Supplementary Fig. 29. XPS spectra of Fe 2*p* for spent 1Mo9FeAl. XPS spectra of Fe 2*p* for 1Mo9FeAl at different reaction time. The a.u. stands for arbitrary units.

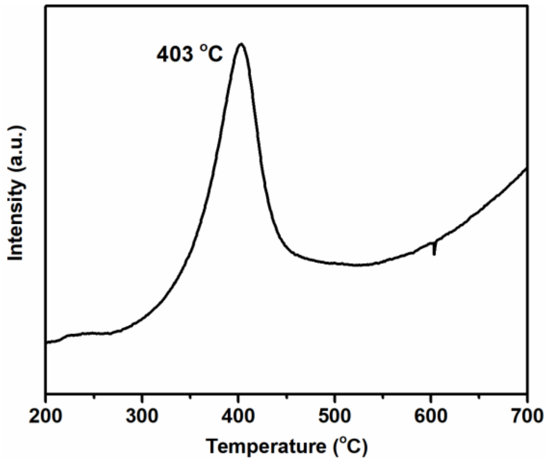

Supplementary Fig. 30. H_2-TPR profile of MoAl. H_2-TPR was performed from 200 °C to 700 °C with a rate of 10 °C/min in a mixture of 10 vol% H_2 in Ar (30 mL/min). The a.u. stands for arbitrary units.

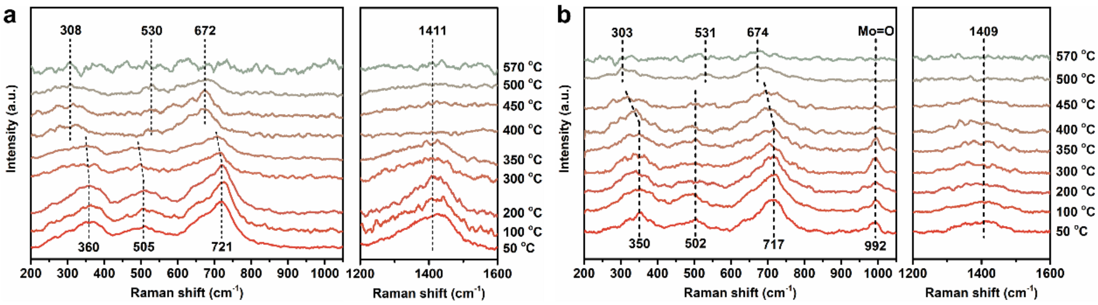

Supplementary Fig. 31. *In situ* Raman spectra of MoO_3-Fe_2O_3 redox catalysts. *In situ* Raman spectra of (a) FeAl and (b) 1Mo9FeAl under the flow of 15 vol% C_3H_8 in He (20 mL/min) at different temperatures. The a.u. stands for arbitrary units.

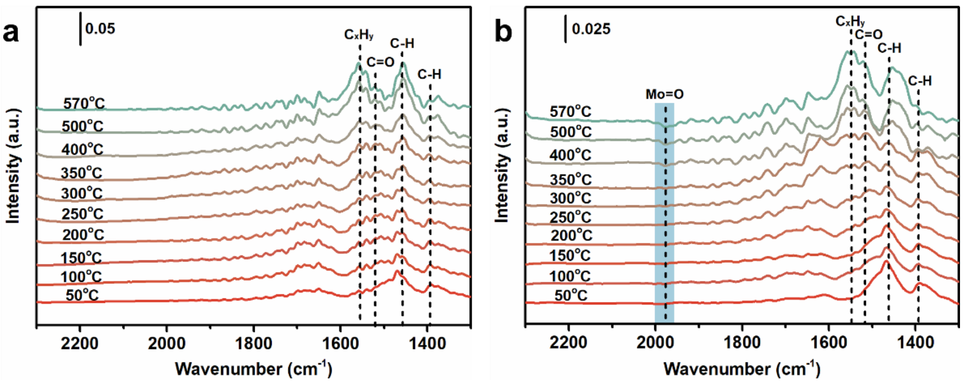

Supplementary Fig. 32. *In situ* DRIFTS spectra of MoO_3-Fe_2O_3 redox catalysts. *In situ* DRIFTS spectra of (a) FeAl and (b) 1Mo9FeAl under the flow of 15 vol% C_3H_8 in He (10 mL/min) at different temperatures. The a.u. stands for arbitrary units.

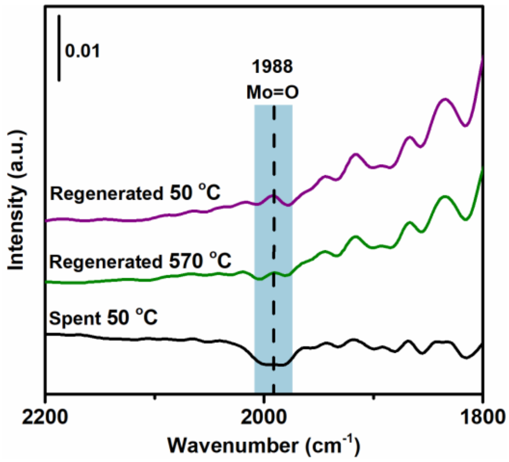

Supplementary Fig. 33. *In situ* DRIFTS spectra of regenerated 1Mo9FeAl. *In situ* DRIFTS spectra of 1Mo9FeAl after regeneration under N_2 flow. The a.u. stands for arbitrary units.

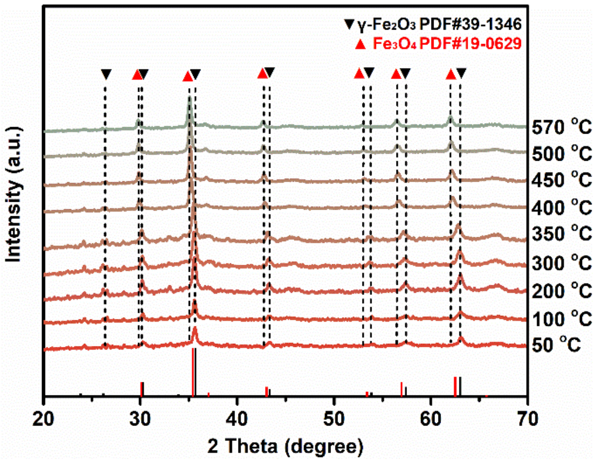

Supplementary Fig. 34. *In situ* XRD patterns of FeAl. *In situ* XRD patterns of FeAl under the flow of 15 vol% C_3H_8 in He (20 mL/min) at different temperatures. The a.u. stands for arbitrary units.

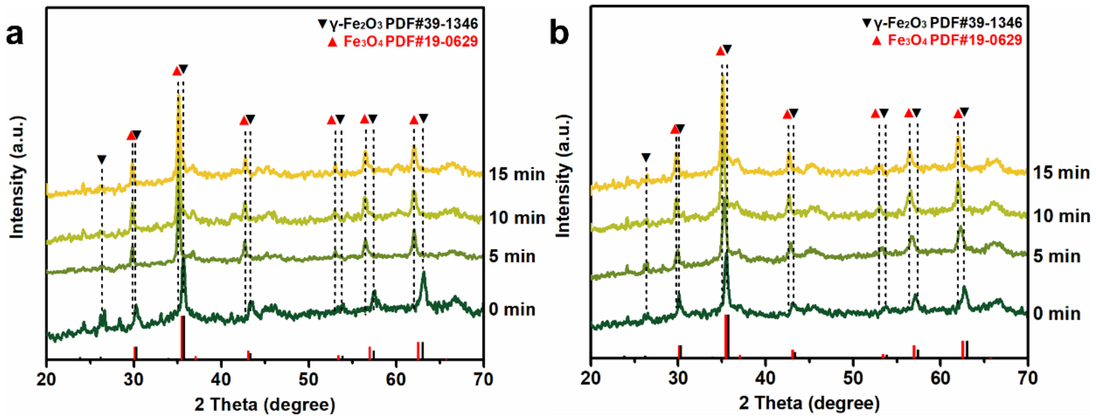

Supplementary Fig. 35. Isothermal *in situ* XRD patterns of MoO_3-Fe_2O_3 redox catalysts. *In situ* XRD patterns of (a) FeAl and (b) 1Mo9FeAl under the flow of 15 vol% C_3H_8 in He (20 mL/min) at 570 °C. The a.u. stands for arbitrary units.

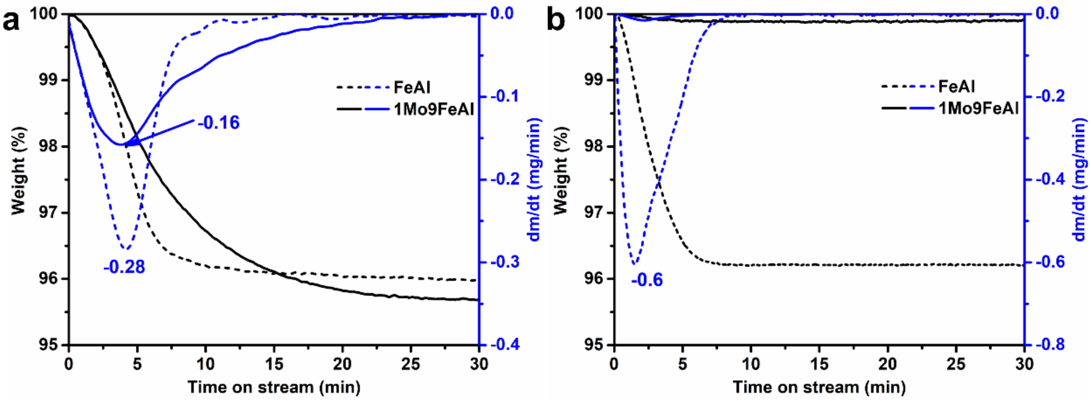

Supplementary Fig. 36. TG and DTG profiles of MoO_3-Fe_2O_3 redox catalysts. TG and DTG profiles of FeAl and 1Mo9FeAl under 15 vol% (a) C_3H_8 and (b) C_3H_6 in He flow (50 mL/min) at 570 °C. The dash line represents the FeAl and the solid line represents 1Mo9FeAl.

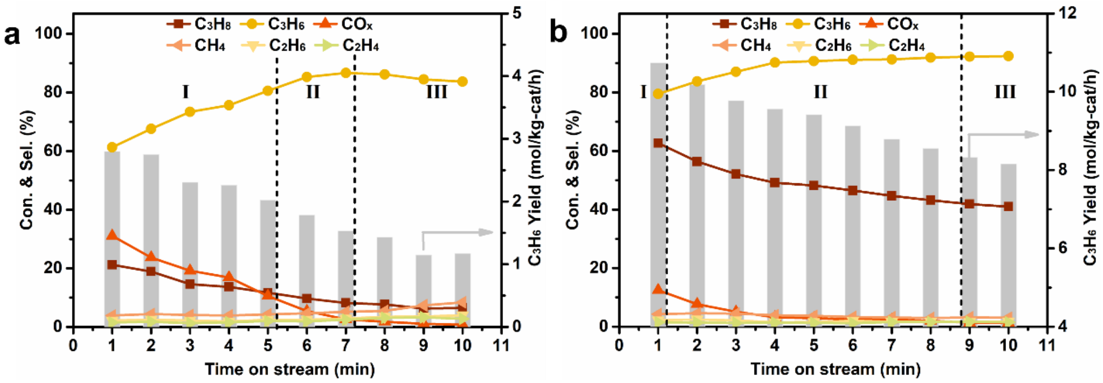

Supplementary Fig. 37. Products distribution over MoO_3-Fe_2O_3 redox catalysts. Products distribution over (a) FeAl and (b) 1Mo9FeAl at different reaction time. Reaction conditions: 570 °C, 0.14 MPa, GHSV = 3000 h^{-1}, 0.5 g sample, volumetric C_3H_8/N_2 ratio = 4:17.

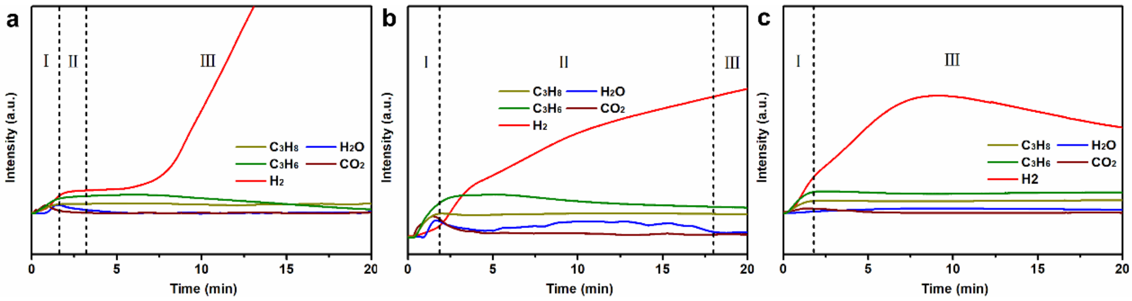

Supplementary Fig. 38. Isothermal reduction of MoO_3-Fe_2O_3 redox catalysts. Mass spectra of H_2, H_2O, CO_2, C_3H_6, C_3H_8 from chemical looping oxidative dehydrogenation of propane over (a) FeAl (b) 1Mo9FeAl and (c) MoAl under the flow of 15 vol% C_3H_8 in He (20 mL/min) at 570 °C. The a.u. stands for arbitrary units.

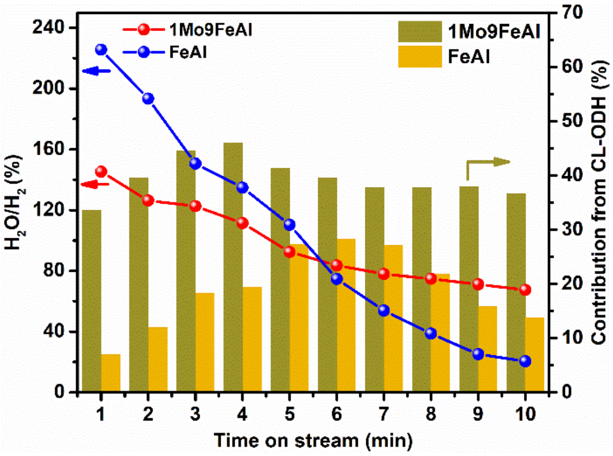

Supplementary Fig. 39. H_2O/H_2 ratio for MoO_3-Fe_2O_3 redox catalysts. H_2O/H_2 ratio at different reaction time over FeAl and 1Mo9FeAl. Reaction conditions: 570 °C, 0.14 MPa, GHSV = 3000 h^{-1}, 0.5 g sample, volumetric C_3H_8/N_2 ratio = 4:17.

Supplementary Table 1. Catalytic performance of reported Fe-based catalysts.

No	Sample	Con. (%)	Sel. (%)	Yie. (%)	Ref.
1	Fe-ZSM-5	7	78	5	4
2	3FeP/Al_2O_3	15	80	12	5
3	Fe-ZSM-5	15	40	6	6
4	Fe-MFI	28	90	25	7
5	Fe-HZSM-5	30	54	16	8
6	20Fe/5S-Al_2O_3	24	79	19	9
7	Fe-ZSM-5-G	14	35	5	10
8	20FeAl-N	14	68	10	11
9	10FeAl	24	83	20	12
10	Fe1-SiO_2	5	99	5	13
11	FeC_600	1	90	1	14
	FeAl	14	76	11	This work
	1Mo9FeAl	49	90	44	This work

Supplementary Table 2. Catalytic performance of reported ODH catalysts.

No	Sample	Con. (%)	Sel. (%)	Yie. (%)	Ref.
1	h-BN	14	79	11	15
2	h-BN	12	70	8	16
3	B_2O_3/CNT	5	50	2	17
4	B_2O_3/SBA-15	32	64	20	18
5	B_1-MFI	41	81	33	19
6	SiB	19	80	15	20
7	MoO_x/Al_2O_3	32	27	9	21
8	Cr_2O_3/SBA-15	30	16	5	22
9	$PdO/Mg_3V_2O_8$	13	58	8	23
10	$Pt/SnO_2/Al_2O_3$	7	84	6	24
11	(Pt/Al_2O_3)@35cIn_2O_3	57	80	46	25
12	Fe_3Ni	3	58	2	26
13	VO_x/CaO-γAl_2O_3	25	94	24	27
14	Mo-V-O	36	89	32	28
15	$LaNiO_x$	10	60	6	29
16	$LaNiO_x$	11	40	4	30
	FeAl	14	76	11	This work
	1Mo9FeAl	49	90	44	This work

Supplementary Table 3. Textural properties of cycled 1Mo9FeAl.

Sample	Crystal size (nm)[a]
1Mo9FeAl-Fresh	25.3
1Mo9FeAl-1 c	26.6
1Mo9FeAl-20 c	29.1
1Mo9FeAl-50 c	29.5
1Mo9FeAl-100 c	30.2
1Mo9FeAl-200 c	30.1
1Mo9FeAl-300 c	31.3

[a] The crystal sizes are calculated from the XRD patterns based on the Scherrer Formula

Supplementary Table 4. XPS derived surface composition of cycled 1Mo9FeAl.

Sample	Fe/Mo_{XPS}[a]
1Mo9FeAl-Fresh	2.9
1Mo9FeAl-100 c	2.9
1Mo9FeAl-200 c	3.3
1Mo9FeAl-300 c	3.5

[a] Fe/Mo_{XPS} is given by XPS analysis.

Supplementary Table 5. Physical absorption results for MoO_3-Fe_2O_3 redox catalysts.

Sample	S_{BET} [m^2/g][a]	V_{total} [cm^3/g][b]
FeAl	35.91	0.22
1Mo15FeAl	35.84	0.23
1Mo12FeAl	35.76	0.22
1Mo9FeAl	35.71	0.22
1Mo6FeAl	35.73	0.21

[a] Total surface area was obtained from multipoint Brunauer-Emmett-Teller (BET) method

[b] Total pore volume

Supplementary Table 6. EXAFS fitting parameters at Mo K-edge.

Sample	Scattering Path	CN [a]	R (Å) [b]	$\sigma^2 \times 10^{-3}$ ($Å^2$) [c]	ΔE (eV) [d]	R factor[e]
Mo foil	Mo-Mo	8*	2.72	4.2	−6.2	0.001
	Mo-Mo	6*	3.15	4.3	−3.2	0.001
1Mo9FeAl	Mo-O	3.8	1.80	2.0	−5.1	0.013
MoO_2	Mo-O	7.2	2.00	5.5	−3.7	0.020
	Mo-O	9.8	3.21	13.8	−3.7	0.020
	Mo-Mo	7.9	3.63	4.3	−9.2	0.020
MoO_3	Mo-O	2.7	1.70	3.7	−6.9	0.055
	Mo-O	1.9	1.94	1.1	−6.9	0.055
	Mo-Mo	4.6	3.68	4.0	−17.0	0.055

[a] CN, coordination numbers;

[b] R, distance between absorber and backscattered atoms.

[c] Change in the Debye-Waller factor value relative to the Debye-Waller factor of the reference compound;

[d] Inner potential correction to account for the difference in the inner potential between the sample and the reference compound;

[e] R factor, goodness of fit;

[*] Fitting with fixed parameter.

Supplementary Table 7. Textural properties of MoO_3-Fe_2O_3 redox catalysts.

Sample	2 Theta (degree)	Lattice constant (nm)[a]	Crystal size (nm)[b]
FeAl	35.79	0.2507	27.9
1Mo15FeAl	35.82	0.2503	26.8
1Mo12FeAl	35.85	0.2499	26.1
1Mo9FeAl	35.89	0.2495	25.3
1Mo6FeAl	35.92	0.2492	23.9

[a] The lattice constant are calculated from the XRD patterns.

[b] The crystal sizes are calculated from the XRD patterns based on the Scherrer Formula

Supplementary Table 8. XPS derived surface composition for MoO_3-Fe_2O_3 redox catalysts.

Sample	Fe/Mo_{ICP}[a]	Fe/Mo_{XPS}[b]
FeAl	-	-
1Mo15FeAl	14.1	4.2
1Mo12FeAl	10.8	3.5
1Mo9FeAl	8.5	2.9
1Mo6FeAl	6.2	2.4

[a] F/Mo_{ICP} is given by ICP analysis. [b] Fe/Mo_{XPS} is given by XPS analysis.

Supplementary Table 9. Fitted NH_3-TPD results for MoO_3-Fe_2O_3 redox catalysts.

Samples	T_D (°C)			total area (a.u.)	Peak area (a.u.)			fitted parameter, R^2
	I	II	III		I	II	III	
Al_2O_3	158	224	401	195	19	30	146	0.99
FeAl	157	220	380	239	38	51	150	0.99
1Mo9FeAl	161	223	382	341	61	127	153	0.99
MoAl	165	226	351	468	71	134	263	0.99

As shown in Fig. 4a, The NH_3-TPD profiles exhibited three desorption peaks (T_D) in the region of 120-200 °C, 200-350 °C and 350-450 °C, ascribed to weak, medium, and strong acid sites, respectively. Semi-quantitative results of total and distribution of different acid species were calculated according to the Gaussian peak fitting method, and the fitted results for deconvoluted peaks are summarized in Table S8.

Supplementary Table 10. XPS derived Mo^{4+} percentage for spent 1Mo9FeAl.

Reaction time (min)	$Mo^{4+}/(Mo^{4+}+Mo^{6+})$ (%)
0	0
1	6.5
2	10.8
4	11.2
8	11.7

Supplementary Table 11. XPS derived Mo^{4+} percentage for spent MoAl.

Reaction time (min)	$Mo^{4+}/(Mo^{4+}+Mo^{6+})$ (%)
0	0
1	14.1
2	17.1
4	19.5
8	23.2

Supplementary Table 12. H_2-TPR data of MoO_3-Fe_2O_3 redox catalysts.

Sample	T_α[a] (°C)	T_β[b] (°C)
FeAl	-	350
1Mo15FeAl	363	444
1Mo12FeAl	371	445
1Mo9FeAl	376	451
1Mo6FeAl	385	463

[a, b] T_α and T_β are the temperature of α and β peaks, respectively given by Gaussian fitting.

Supplementary Table 13. Elimination of internal and external mass transfer limitation.

Parameter	Value	Notes
D (μm)	230	Maximum diameter was used to consider the worse-case scenario.
D_{AB} (m^2/s)	7.5×10^{-5}	Estimated using the method in Bird et al.[31]
D_{eff} (m^2/s)	7.5×10^{-6}	Estimated as $D_{eff} = 0.1D_{AB}$.[32]
$C_{C_3H_8b}$ (mol/m^3)	0.290	Estimated using ideal gas law at P = 1 atm and T = 843 K (570 °C) for 2 Vol% C_3H_8
$C_{C_3H_8s}$ (mol/m^3)	0.290	Estimated using $C_{C_3H_8b}$ in order to consider the worse-case scenario
$\rho_{1Mo9FeAl}$ (kg/m^3)	4500	
ϕ	0.4	
n	0.80	
$-r'_{C_3H_8obs}$ (mol/g-cat s)	1.96×10^{-4}	Highest observed rate was used.
MR	3.89×10^{-3}	MR << 0.15, no external mass transfer limitation
C_{WP}	5.36×10^{-3}	C_{WP} << 1, no internal mass transfer limitation

Supplementary Table 14. Kinetics coefficients for the reduction of FeAl and 1Mo9FeAl

Sample	*k* (cm/s)	*D* (cm^2/s)
FeAl	$1.2 * 10^{-5}$	$2.5 * 10^{-8}$
1Mo9FeAl	$1.6 * 10^{-4}$	$6.7 * 10^{-9}$

Supplementary References

[1] J. Tang, M. Myers, K. A. Bosnick, L. E. Brus, *J. Phys. Chem. B* **2003**, *107*, 7501-7506.

[2] S. Yu, G. M. Chow, *J. Mater. Chem.* **2004**, *14*, 2781-2786.

[3] J. H. Hsu, P. C. Kuo, C. W. Hsu, *J. Appl. Phys.* **1990**, *67*, 5152-5154.

[4] J. H. Yun, R. F. Lobo, *J. Catal.* **2014**, *312*, 263-270.

[5] S. Tan, B. Hu, W.-G. Kim, S. H. Pang, J. S. Moore, Y. Liu, R. S. Dixit, J. G. Pendergast, D. S. Sholl, S. Nair, C. W. Jones, *ACS Catal.* **2016**, *6*, 5673-5683.

[6] A. Ates, C. Hardacre, A. Goguet, *Appl. Catal. A* **2012**, *441-442*, 30-41.

[7] G. Wu, F. Hei, N. Guan, L. Li, *Catal. Sci. Technol.* **2013**, *3*, 1333.

[8] P. Sazama, N. K. Sathu, E. Tabor, B. Wichterlová, Š. Sklenák, Z. Sobalík, *J. Catal.* **2013**, *299*, 188-203.

[9] Y.-n. Sun, L. Tao, T. You, C. Li, H. Shan, *Chem. Eng. J.* **2014**, *244*, 145-151.

[10] G. Wu, Y. Hao, N. Zhang, N. Guan, L. Li, W. Grünert, *Micropor. Mesopor. Mater.* **2014**, *198*, 82-91.

[11] Y. Sun, Y. Wu, H. Shan, G. Wang, C. Li, *Catal. Sci. Technol.* **2015**, *5*, 1290-1298.

[12] Y. Sun, Y. Wu, L. Tao, H. Shan, G. Wang, C. Li, *J. Mol. Catal. A* **2015**, *397*, 120-126.

[13] B. Hu, N. M. Schweitzer, G. Zhang, S. J. Kraft, D. J. Childers, M. P. Lanci, J. T. Miller, A. S. Hock, *ACS Catal.* **2015**, *5*, 3494-3503.

[14] M. L. Sarazen, C. W. Jones, *J. Phys. Chem. C* **2018**, *122*, 28637-28644.

[15] J. T. Grant, C. A. Carrero, F. Goeltl, J. Venegas, P. Mueller, S. P. Burt, S. E. Specht, W. P. McDermott, A. Chieregato, I. Hermans, *Science* **2016**, *354*, 1570-1573

[16] J. Tian, J. Tan, M. Xu, Z. Zhang, S. Wan, S. Wang, J. Lin, Y. Wang, *Sci. Adv.* **2020**, *5*, eaav8063.

[17] B. Frank, J. Zhang, R. Blume, R. Schlogl, D. S. Su, *Angew. Chem. Int. Ed.* **2009**, *48*, 6913-6917.

[18] W.-D. Lu, D. Wang, Z. Zhao, W. Song, W.-C. Li, A.-H. Lu, *ACS Catal.* **2019**, 8263-8270.

[19] H. Zhou, X. Yi, Y. Hui, L. Wang, W. Chen, Y. Qin, M. Wang, J. Ma, X. Chu, Y. Wang, X. Hong, Z. Chen, X. Meng, H. Wang, Q. Zhu, L. Song, A. Zheng, F.-S. Xiao, *Science* **2021**, *372*, 76-80.

[20] B. Yan, W.-C. Li, A.-H. Lu, *J. Catal.* **2019**, *369*, 296-301.

[21] M. C. Abello, M. F. Gomez, O. Ferretti, *Appl. Catal. A* **2012**, *207*, 421-431.

[22] X. Zhang, Y. Yue, Z. Gao, *Catal. Lett.* **2002**, *83*, 19-25.

[23] S. Sugiyama, Y. Hirata, K. Nakagawa, K. I. Sotowa, K. Maehara, Y. Himeno, W. Ninomiya, *J. Catal.* **2008**, *260*, 157-163.

[24] S. Vajda, M. J. Pellin, J. P. Greeley, C. L. Marshall, L. A. Curtiss, G. A. Ballentine, J. W. Elam, S. Catillon-Mucherie, P. C. Redfern, F. Mehmood, P. Zapol, *Nat. Mater.* **2009**, *8*, 213-216.

[25] H. Yan, K. He, I. A. Samek, D. Jing, M. G. Nanda, P. C. Stair, J. M. Notestein, *Science* **2021**, *371*, 1257-1260.

[26] E. Gomez, S. Kattel, B. Yan, S. Yao, P. Liu, J. G. Chen, *Nat. Commun.* **2018**, *9*, 1398.

[27] M. M. Hossain, *Ind. Eng. Chem. Res.* **2017**, *56*, 4309-4318.

[28] S. Chen, L. Zeng, R. Mu, C. Xiong, Z. J. Zhao, C. Zhao, C. Pei, L. Peng, J. Luo, L. S. Fan, J. Gong, *J. Am. Chem. Soc.* **2019**, *141*, 18653-18657.

[29] S. Crapanzano, I. V. Babich, L. Lefferts, *Appl. Catal. A* **2010**, *385*, 14-21.

[30] S. Crapanzano, I. V. Babich, L. Lefferts, *Appl. Catal. A* **2011**, *391*, 70-77.

[31] R. B. Bird, W. E. Stewart, E. N. Lightfoot, D. J. Klingenberg, *Introductory transport phenomena*, Wiley, Hoboken, NJ, **2015**, pp 508-512.

[32] K. M. Hardiman, C. G. Cooper, A. A. Adesina, R. Lange, *Chem. Eng. Sci.* **2006**, *61*, 2565-2573.
